# A Zwitterionic Heterobimetallic Gold–Iron Complex Supported by Bis(*N*‐Heterocyclic Imine)Silyliumylidene

**DOI:** 10.1002/anie.202108146

**Published:** 2021-09-22

**Authors:** Franziska Hanusch, Dominik Munz, Jörg Sutter, Karsten Meyer, Shigeyoshi Inoue

**Affiliations:** ^1^ Department of Chemistry Catalysis Research Center and Institute of Silicon Chemistry Technische Universität München (TUM) Lichtenbergstrasse 4 85748 Garching bei München Germany; ^2^ Faculty of Natural Sciences and Technology Inorganic Chemistry: Coordination Chemistry Saarland University Campus C4 1 66123 Saarbrücken Germany; ^3^ Department of Chemistry and Pharmacy Inorganic Chemistry Friedrich-Alexander-Universität Erlangen-Nürnberg (FAU) Egerlandstrasse 1 91058 Erlangen Germany

**Keywords:** anagostic interactions, gold, iron, silicon, zwitterionic complexes

## Abstract

The facile synthesis of the first bis‐*N*‐heterocyclic imine‐stabilized chlorosilyliumylidene **1** is reported. Remarkably, consecutive reaction of **1** with PPh_3_AuCl and K_2_Fe(CO)_4_ gives rise to the unique heterobimetallic complex 1,2‐(^Mes^NHI)_2_‐C_2_H_4_‐ClSiAuFe(CO)_4_ (**4**). The overall neutral complex **4** bears an unusual linear Si−Au−Fe structure and a rare anagostic interaction between the *d* ^10^‐configured gold atom and a CH bond of the mesityl ligand. According to the computational analysis and ^57^Fe Mössbauer spectroscopy, the formal Fe‐oxidation state remains at −II. Thus, the electronic structure of **4** is best described as an overall neutral—yet zwitterionic—heterobimetallic “Si(II)^+^‐Au(I)^+^‐Fe(‐II)^2−^”‐silyliumylidene complex, derived from double anion exchange. The computational analysis indicates strong hyperconjugative back donation from the gold(I) atom to the silyliumylidene ligand.

## Graphical abstract

The selective synthesis of a silyliumylidene‐stabilized mononuclear Au−Fe complex, by a convenient one‐pot reaction starting from bis‐NHI silyliumylidene chloride, is reported. X‐ray crystallography, IR and ^57^Fe Mössbauer spectroscopy, as well as computations suggest a zwitterionic Si(II)^+^−Au(I)^+^−Fe(‐II)^2−^ bonding motif with rare anagostic CH⋅⋅⋅Au contacts and considerable backbonding capabilities of the silyliumylidene.

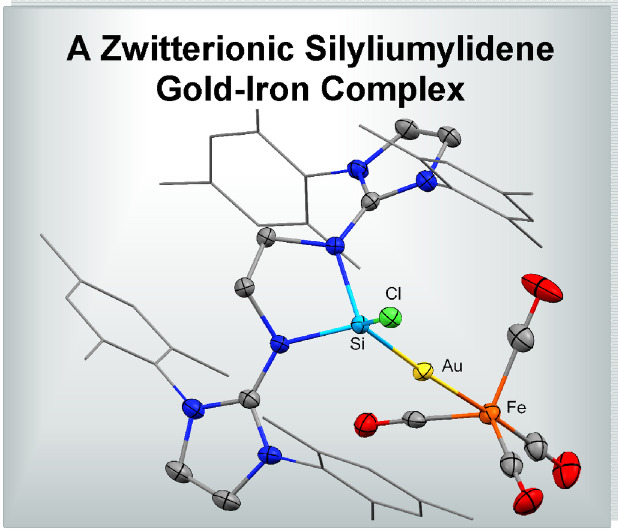

## Introduction

**Figure 1:**
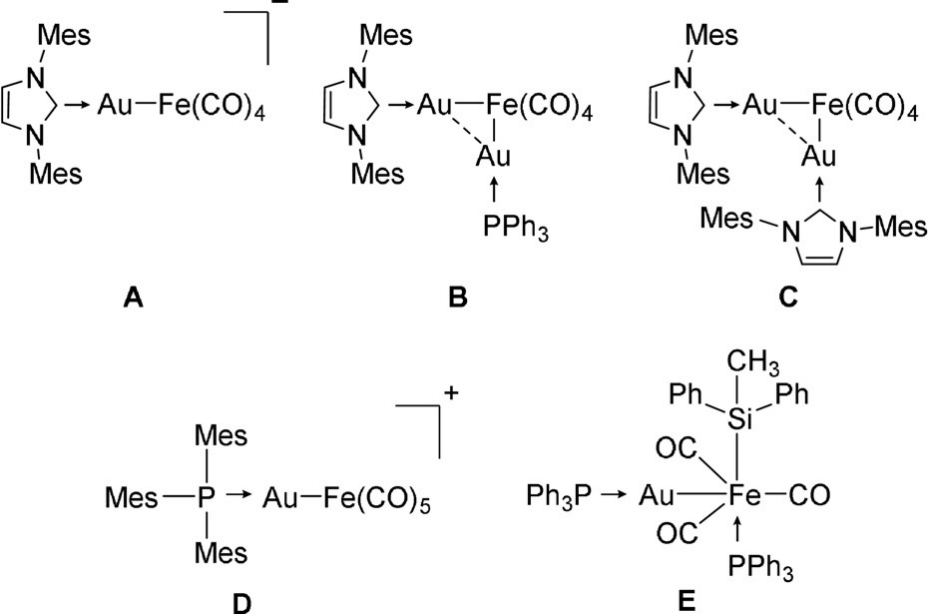
Selected examples for ligand stabilized monomeric (**A**, **D**, **E**) and dimeric (**B**, **C**) heterobimetallic Au‐Fe complexes (Mes=2,4,6‐trimethylphenyl).

Iron and gold engage in strong metallophilic interactions^[1]^ and show—both in homogeneous and heterogeneous phase—remarkable catalytic activity in industrial relevant processes, such as valorization of CO or hydrogenation of olefins.^[2]^ However, heterogeneous catalysts, including active clusters and nanoparticles, often suffer from agglomeration, non‐uniform size distribution, and alloy segregation.^[2a]^ Thus, the identification of novel preparative building blocks that ensure the ideal mixing of the alloy metals is of significant current importance. Heterobimetallic complexes arguably represent monomeric subunits of alloy clusters and feature a bifunctional metal core. Cooperativity between these two well‐defined sites is believed to engender unusual chemical transformations, and may serve as models; hence, improving our understanding of heterogeneous reaction mechanisms.^[3]^ Still, defined small clusters or even heterobimetallic complexes with a monomeric Au−Fe unit are rare.[^1a^, ^2b^, ^4^] So far, sterically demanding donor ligands, such as NHCs (*N*‐heterocyclic carbenes) or phosphines, were applied for the isolation of these smallest units of the gold‐iron alloy (Figure 1).^[5]^

**Figure 2:**
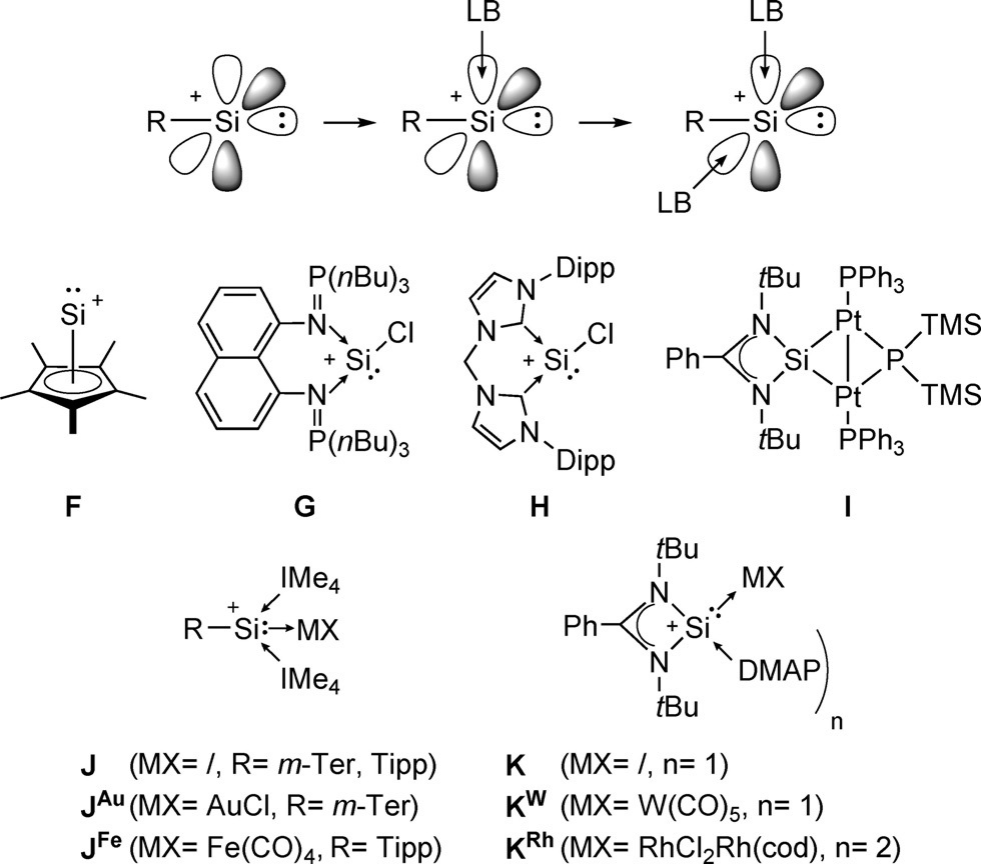
Schematic representation of silyliumylidene cations (R=covalent bound substituent; LB=Lewis base), and selected examples for reported silyliumylidene ions, and silyliumylidene ion metal complexes (*m*‐Ter=2,6‐mesitylphenyl, Dipp=2,6‐diisopropylphenyl, Tipp=2,4,6‐triisopropylphenyl, DMAP=4‐dimethylaminopyridine).

Silicon's ability to partially mimic its lighter congener carbon, and to show metal‐like behavior at the same time, has been a powerful concept for small molecule activation and preparation of novel materials.^[6]^ In recent years, silicon‐based ligands beyond conventional silyl‐ligands have been highlighted. Besides the nowadays well‐known silylene ligands,^[7]^ silyliumylidenes are equally fascinating; yet, remain comparatively unexplored.^[8]^ Silyliumylidenes can serve as ligands in transition metal chemistry through their accessible lone pair of electrons, and their cationic nature offers coordination chemistry complementary to silylenes (Figure 2).^[9]^

Hitherto known silyliumylidene metal complexes are mono‐ (**J^Au^**, **J^Fe^**, **K^W^**) or homobimetallic (**I**, **K^Rh^**) but, so far, no heterobimetallic silyliumylidene complex is reported.^[10]^ Even in silylene chemistry, examples are scarce and are mainly represented by bissilylenes or other multinuclear silylene systems.^[11]^ Due to the unique electronic properties of silyliumylidenes, we hypothesize them to be promising candidates to stabilize uncommon bonding motifs, such as monomeric heterobimetallic Au−Fe complexes.[^10a^, ^10b^, ^10c^, ^12^] Recently, we have shown that bis‐NHIs (bis‐*N*‐heterocyclic imines/ bis‐imidazoline‐2‐imines)^[13]^ are suitable for the stabilization of electron deficient main group complexes.^[14]^ Hence, we expect the strong donor abilities in combination with the chelating effect of bridged bis‐NHIs to provide a significant advantage in the formation of reactive silyliumylidenes and their metal complexes.[^13a^, ^15^] Herein, we present the synthesis of a bis‐NHI‐stabilized silyliumylidene ion and its reactivity towards the heavier chalcogens and coinage metals. Furthermore, an overall neutral, heterobimetallic silyliumylidene‐gold complex bearing a coordinated Fe(CO)_4_^2−^ dianion (**4**) is reported for the first time.

## Results and Discussion

**Scheme 1:**
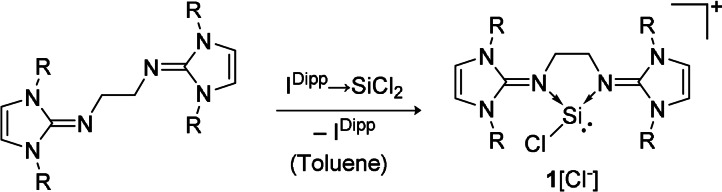
Synthesis of bis‐NHI‐stabilized chlorosilyliumylidene cation **1** (R=mesityl=2,4,6‐trimethylphenyl).

Upon reaction of the neutral ligand 1,2‐(^Mes^NHI)_2_‐C_2_H_4_ (^Mes^NHI=1,3‐bis(mesityl)‐imidazolin‐2‐imine, mesityl=2,4,6‐trimethylphenyl) with an equimolar amount of I^Dipp^‐stabilized dichloro‐silylene^[16]^ in toluene, chlorosilyliumylidene **1** was isolated in 76 % yield as a colorless, pearly solid (Scheme 1). Compound **1** is stable under inert conditions and soluble in acetonitrile, difluorobenzene, and pyridine, whereas it decomposes in chlorinated solvents, such as chloroform and dichloromethane.

**Figure 3:**
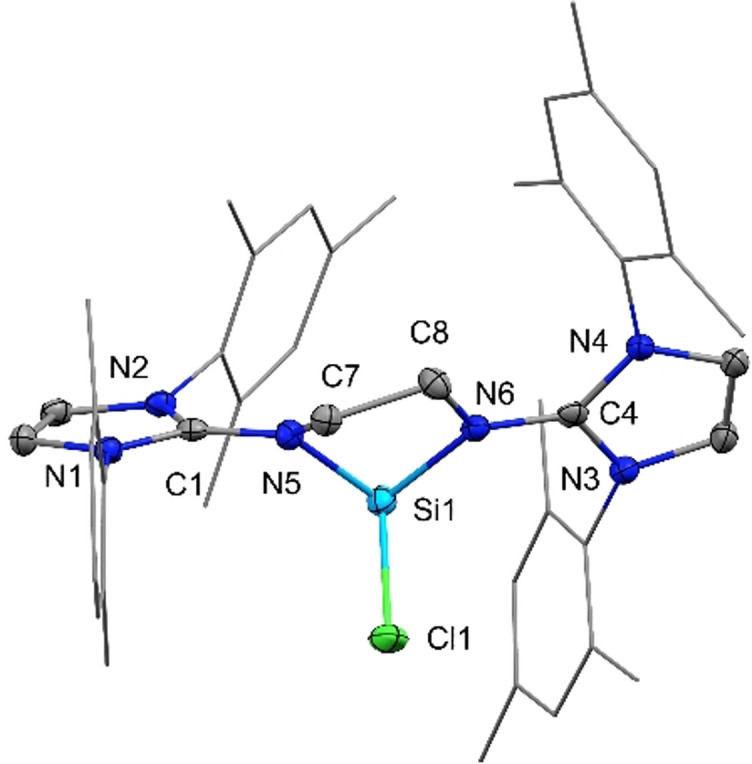
Solid‐state plot of the molecular structure of **1**. Thermal ellipsoids are set at 50 % probability. Hydrogen atoms, the counter anion and solvent molecules are omitted for clarity; mesityl‐substituents are depicted as wireframe for simplicity.^[39]^ Selected bond lengths [Å] and angles [°]: Si1–Cl1 2.2374(8), Si1–N5 1.8290(18), Si1–N6 1.8617(18), C1–N5 1.337(3), C4–N6 1.336(3); Cl1–Si1–N5 95.07(6), N5–Si1–N6 84.00(8), Cl1–Si1–N6 94.91(6).

The ^29^Si NMR resonance for **1** was observed at δ(^29^Si)=+1.5 ppm, which is comparable to bis(iminophosphorane)‐stabilized silyliumylidene **G** ^[10e]^ (δ(^29^Si)=−3.3 ppm). This indicates a similar coordination environment as well as a comparable electronic structure. In contrast, the ^29^Si NMR resonance in NHC‐stabilized silyliumylidenes **H** ^[12]^ (δ(^29^Si)=−58.4 ppm), **J**[^8a^, ^10g^] (δ(^29^Si)=−68.8 ppm), or the amidinate‐type stabilized silyliumylidene **K** ^[10f]^ (δ(^29^Si)=−82.3 ppm) are shifted to higher fields. The structural similarity to **G** was confirmed by single‐crystal X‐ray diffraction (SC‐XRD) analysis of colorless crystals grown from an *ortho*‐difluorobenzene/ diethylether solution. Compound **1** crystalizes in the triclinic space group *P*−1 with the Si‐atom in trigonal pyramidal geometry and the sum of bond angles around silicon being 273.9(2)° (Figure 3). The coordinated chlorine Cl1 protrudes from the puckered 5‐membered C_2_N_2_Si‐ring at an angle of ca. 90°.

**Scheme 2:**
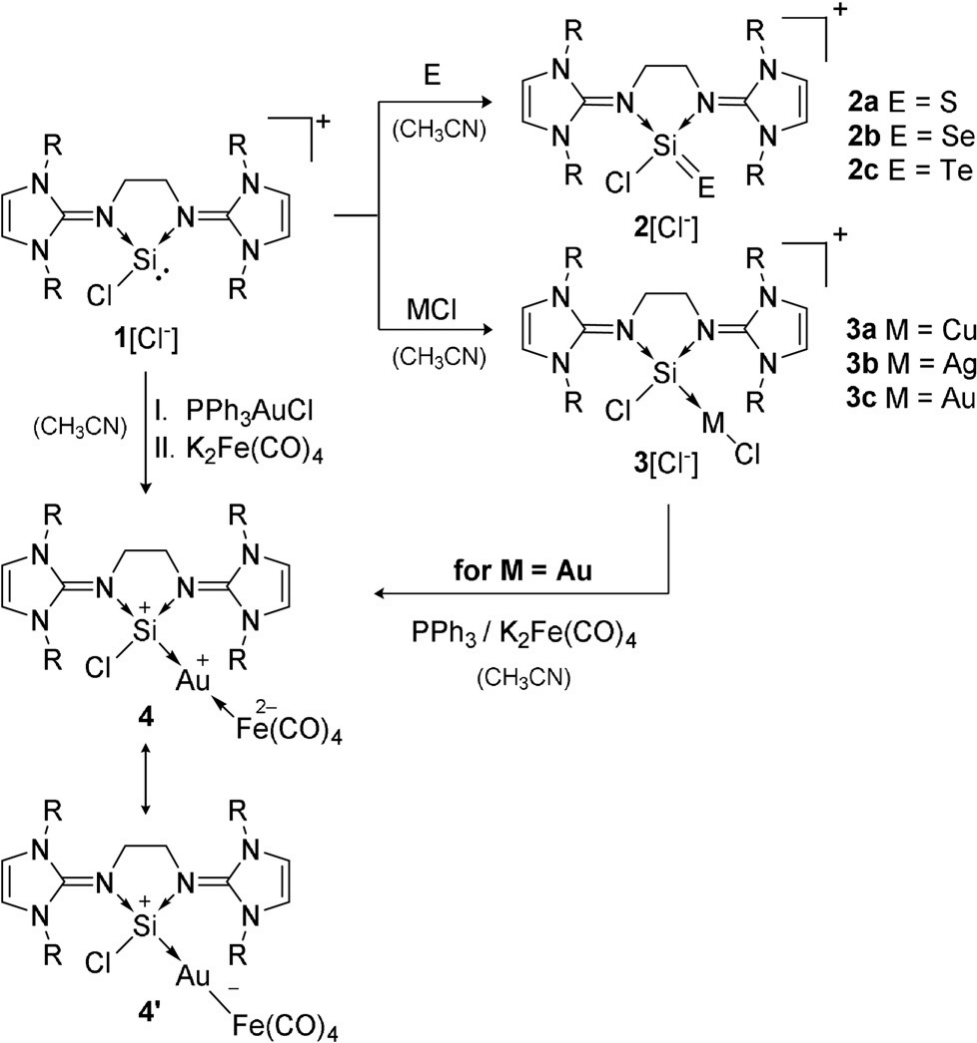
Conversion of bis‐NHI stabilized silyliumylidene **1** (R=mesityl=2,4,6‐trimethylphenyl) into heavier silaacylium ions **2** (E=S, Se, Te), coinage metal complexes **3** (MCl=CuCl, AgCl, (Me_2_S)AuCl), and novel heterobimetallic silyliumylidene complex **4**.

With silyliumylidene **1** in hand, we investigated the reactivity of its lone pair of electrons, by oxidation with heavier chalcogenes. Indeed, elemental sulfur, selenium, and tellurium could be activated by stirring with **1** in acetonitrile at room temperature, furnishing the heavier silaacylium ions **2 a**–**2 c** in nearly quantitative yields (Scheme 2, top reaction pathway).

While sulfur reacted readily within 30 minutes to give **2 a**, the reaction took 48 hours in case of tellurium for complete conversion to **2 c**. Driess et al. proved **G** to be capable of sulfur activation to form the silathionium cation **G^S^** ^[10e]^ and similar chalcogen activations were found for **J** and **K**.[^10f^, ^17^] The ^29^Si NMR spectroscopic analysis revealed the expected trend of upfield shift from **2 a** to **2 c** with δ(^29^Si)=−26.7 ppm (**2 a**), −31.0 ppm (**2 b**), −59.1 ppm (**2 c**), that was also observed for the heavier silaacylium ions of **J**. For bis(iminophosphorane) stabilized **G^S^**, a sharp singlet at −26.7 ppm was observed.^[10e]^The heteronuclear NMR shifts of **2 b** (*δ*(^77^Se)=−388.8 ppm) and **2 c** (*δ*(^125^Te)=−1049.4 ppm) are within the range of known three‐coordinate Si=E complexes (E=Se: −244 to −655 ppm;^[18]^ E=Te: −738 to −1264 ppm).^[19]^

The investigation of ^77^Se NMR resonances is a well‐established method to assess the π‐acidity of NHCs and thus might also be indicative for Si=Se compounds.^[20]^ As anticipated, due to the electronegative chlorine substituent, **2 b** seems to be a stronger π‐acceptor than the *m*‐terphenyl‐substituted selenium adduct **J^Se^** (δ(^77^Se)=−423.8 ppm),^[17]^ and also three‐coordinate Si=Se with anionic *N*,*N*‐chelating ligands and additional donors tend to resonate at higher field.^[21]^ However, compared to chlorine‐substituted NHSi [Ph_2_P(N*t*Bu)_2_]ClSi=Se (δ(^77^Se)=−267.9 ppm)^[18a]^ and, intriguingly, with selenides of commonly used phosphine ligands R_3_P=Se (δ(^77^Se)=−28 to −350 ppm),^[22]^ **2 b** shows an upfield shift, arguably indicating lower π‐acidity of the cationic “ligand” **1**.

**Figure 4:**
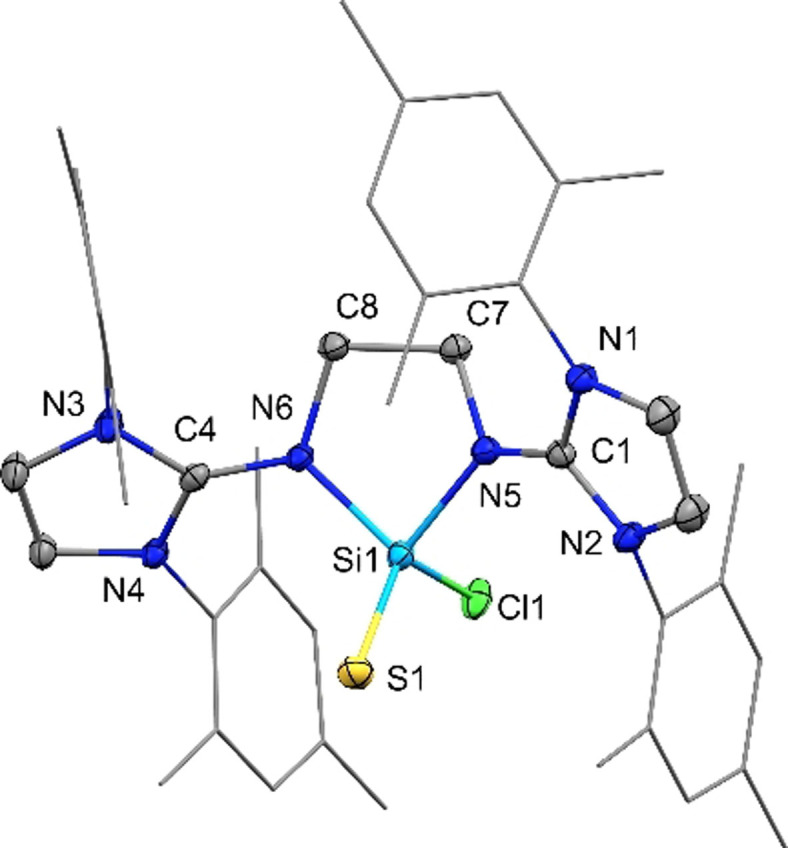
Solid‐state plot of the molecular structure of **2 a**. Thermal ellipsoids are set at 50 % probability. Hydrogen atoms, the counter anion and solvent molecules are omitted for clarity; mesityl‐substituents are depicted as wireframe for simplicity.^[39]^ Selected bond lengths [Å] and angles [°]: Si1–S1 1.9740(6), Si1–Cl1 2.0678(6), Si1–N5 1.7839(12), Si1–N6 1.7972(14), C1–N5 1.3446(19), N6–C4 1.356(2); N5–Si1–N6 89.89(6), N5–Si1–S1 120.22(5), S1–Si1–Cl1 117.05(3), N5–Si1–Cl1 101.96(5).

SC‐XRD analysis of compound **2 a** was carried out on colorless crystals grown from a MeCN/THF mixture. Upon addition of sulfur, the silicon atom adopts a tetrahedral coordination (Figure 4). The Si=S bond length (1.9740(6) Å) is in good agreement with other donor stabilized Si=S double bonds (1.96–2.08 Å)[^17^, ^21a^, ^23^] reported before and matches the Si=S bond in **G^S^** (1.984(2) Å). The molecular structure of **2 a** serves as a representative for compounds **2** by replacing S for Se or Te. Compounds **2 b** and **2 c** were characterized by multinuclear NMR techniques, including ^77^Se (for **2 b**) and ^125^Te (for **2 c**) as well as mass spectrometry (ESI). All data are consistent and, therefore, suggest that **2 b** and **2 c** are isostructural with **2 a**.

Motivated by our work on transition metal complexes of **J**, we also attempted the conversion of **1** with the coinage metal salts Cu^I^Cl, Ag^I^Cl, and Me_2_SAu^I^Cl, respectively (Scheme 2, middle reaction pathway).[^10b^, ^10d^]

Adapting the previously reported experimental procedure,^[10d]^ **1** was treated with an equimolar amount of the precursor (or two equivalents in the case of Ag to ensure complete conversion of poorly soluble precursor salt) at ambient temperature to furnish coinage metal complexes **3 a**–**3 c** as colorless solids in good yields. Complexes **3** could be identified via ^1^H, ^13^C, and ^29^Si NMR techniques and mass spectrometry (ESI). Coordination of the corresponding transition metal causes a significant downfield shift in ^29^Si NMR for **3 a** δ(^29^Si)=+13.8 ppm, **3 b** +20.2 ppm (^1^ *J*_Si_^109^_Ag_=592.8 Hz, ^1^ *J*_Si_^107^_Ag_=514.3 Hz), and **3 c** +18.2 ppm. Compound **3 b** features the up to now highest observed Si−Ag coupling constant with 592.8 Hz for the ^109^Ag nucleus. The shift for the gold complex **3 c** does not follow the expected downfield trend for the series of coinage metal complexes; we attribute this anomaly to relativistic effects.^[24]^

Halogen abstraction reactions using K_2_Fe(CO)_4_ were pursued in order to obtain the targeted heterobimetallic Si−M−Fe complexes (M=Cu, Ag, Au). However, reaction of K_2_Fe(CO)_4_ with **3 a** showed no conversion, even after 16 hours at 60 °C, whereas **3 b** reacted readily under regeneration of **1**. When **3 c** was treated with K_2_Fe(CO)_4_ at low temperature, a mixture of various products was formed that could neither be isolated nor identified. We hypothesized that the addition of an auxiliary donor molecule might stabilize the unsupported vacant coordination site after chloride abstraction. Indeed, adding PPh_3_ to **3 c** prior to K_2_Fe(CO)_4_ resulted in the formation of **4**, which was isolated as bright yellow crystals (Scheme 2, bottom reaction pathway). Notably, **4** is also accessible through one‐pot reaction of **1** with the metal precursor PPh_3_AuCl and subsequent addition of K_2_Fe(CO)_4_. Using this procedure, **4** can be isolated in higher purity and in 54 % yield. Neutral **4** is insoluble in benzene, fluorobenzene, and THF, sparingly soluble in acetonitrile and dissolves readily in pyridine. It is stable in pyridine solution up to 80 °C according to ^1^H NMR monitoring.

**Figure 5:**
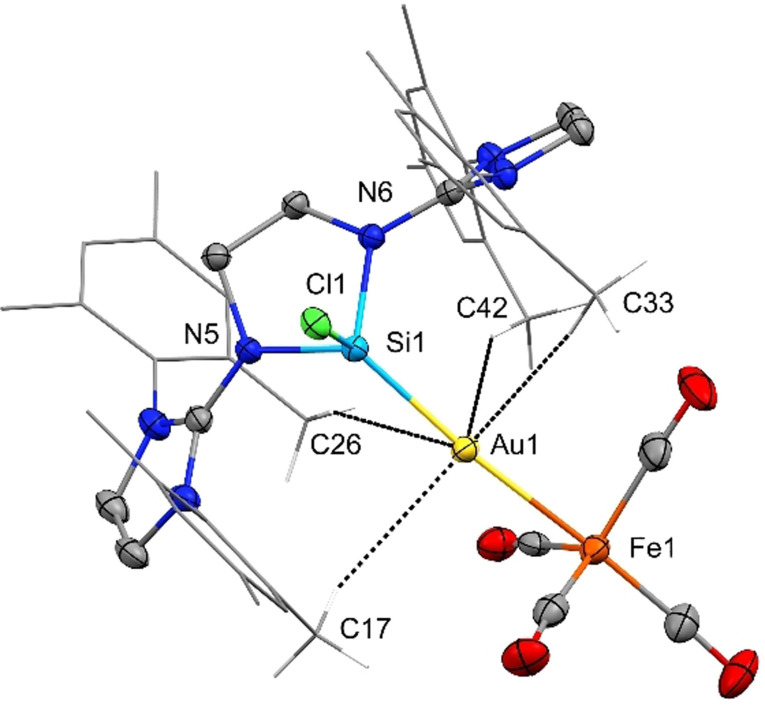
Solid‐state plot of the molecular structure of **4**. Thermal ellipsoids are set at 50 % probability. Hydrogen atoms are omitted for clarity; mesityl‐substituents are depicted as wireframe for simplicity.^[39]^ Selected bond lengths [Å] and angles [°]: Au1–Si1 2.2676(9), Au1–Fe1 2.5305(6), Si1–N5 1.793(3), Si1–N6 1.819(2), Si1–Cl1 2.1076(11), C*H*42⋅⋅⋅Au1 2.70(3), C*H*17⋅⋅⋅Au1 2.87(6), C*H*33⋅⋅⋅Au1 2.97(5), C*H*26⋅⋅⋅Au1 3.58(4); Si1–Au1–Fe1 173.65(3), Cl1–Si1–Au1 119.02(5), N5–Si1–N6 87.80(13) C42–*H*42⋅⋅⋅Au1 156(3), C17–*H*17⋅⋅⋅Au1 127(3), C33–*H*33⋅⋅⋅Au1 167(3), C26–*H*26⋅⋅⋅Au1 161(3).

SC‐XRD analysis confirmed the heterobimetallic structure of **4**. The silicon atom remains in a distorted tetrahedral coordination environment with the N−Si−N angle being strained to 87.80(13)°, as shown in Figure 5. As common for Au^I^ complexes, the gold atom is nearly linearly coordinated with a Si−Au−Fe angle of 173.65(3)°. The Si−Au bond length (2.2676(9) Å) falls within the range of 2.246–2.363 Å for a Si(II)−Au complex.^[25]^ The Au−Fe distance of 2.5305(6) Å matches related compound **A** (2.5168 Å) and is within the range of Au−Fe single bond lengths in small molecular gold‐iron clusters (**A**‐**E**: 2.516–2.567 Å). Moreover, comparatively short C−*H*⋅⋅⋅Au distances can be detected for the mesityl's *ortho*‐methyl groups pointing towards the gold atom, indicating rare anagostic interactions. The shortest contact is detected for C*H*42⋅⋅⋅Au with 2.70(3) Å, while two more are slightly elongated (C*H*17⋅⋅⋅Au 2.87(6), C*H*33⋅⋅⋅Au 2.97(5) Å) but still shorter than Σ_vdW_(H,Au)=3.3 Å.^[26]^ Also computationally, an anagostic interaction is found between the gold ion and a methyl group of the mesityl ligand (BP: 2.361 Å; PBE0: 2.445 Å; Figure 8). Although agostic interactions of Au^III^ complexes have been recently evidenced, C−*H*⋅⋅⋅Au interactions remain rare in general.^[27]^ Also, anagostic C−*H*⋅⋅⋅Au interactions are a topic of current interest.^[28]^ In contrast to agostic interactions that are understood as three‐center‐two‐electron bonds between C−H and vacant *d*‐orbitals of transition metals with rather short H−M distances and narrow C−H−M angles (≈1.8–2.3 Å; ≈90–140°), the weaker anagostic interactions are associated with filled *d*‐orbitals, longer H−M distances and larger C−H−M angles (≈2.3–2.9 Å; ≈110–170°).^[29]^ Consequently, M^I^ (M=Cu, Ag, Au) complexes, that exclusively feature occupied *d*‐orbitals, are good candidates to observe such contacts. The ^29^Si NMR spectrum of **4** displays a single resonance at δ(^29^Si)=+67.6 ppm. This is further downfield shifted compared to starting material **1** (+1.5 ppm) and precursor compound **3 c** (+18.2 ppm). Infrared (IR) vibrational spectroscopic measurements reveal the carbonyl stretching frequencies of **4** to occur at 1924, 1835, 1811, and 1796 cm^−1^ (Table 1). The positions of these bands, which were reproduced by calculations at the ZORA‐BP86‐D3/def2‐SVP level of theory,^[30]^ is indicative for the donor properties, that is, combined σ‐donor/π‐acceptor abilities of the “ligand” **3 c**, which coordinates anionic Fe(CO)_4_ in **4**. Compared to related compounds (*cf*. **A**, **J^Fe^**, I^Mes^−Fe(CO)_4_, Na_2_Fe(CO)_4_, see Table 1) these bands are shifted to higher wavenumbers in case of zwitterionic **4**.^[31]^

**Table 1:** Experimental (AT‐IR) and computed (ZORA‐BP86‐D3BJ/def2‐SVP) CO stretching frequencies (cm^−1^) of **4** and relevant (gold‐)iron carbonyl compounds.

Compound	Experimental	Calculated
**4** ^[a]^	1924, 1835, 1811, 1796	1954, 1872, 1833, 1825
**A** ^[b]^	1975, 1927, 1830, 1790	1926, 1866, 1843, 1818
**J^Fe^** ^[a]^	2021, 1943, 1903, 1887	2027, 1958, 1915, 1895
I^Mes^‐Fe(CO)_4_^[b]^	2035, 1949, 1915	2022, 1959, 1934, 1920
Na_2_Fe(CO)_4_^[a]^	1762	1787

[a] solid state, neat AT‐IR. [b] solid state, neat, nujol‐IR.

**Figure 6:**
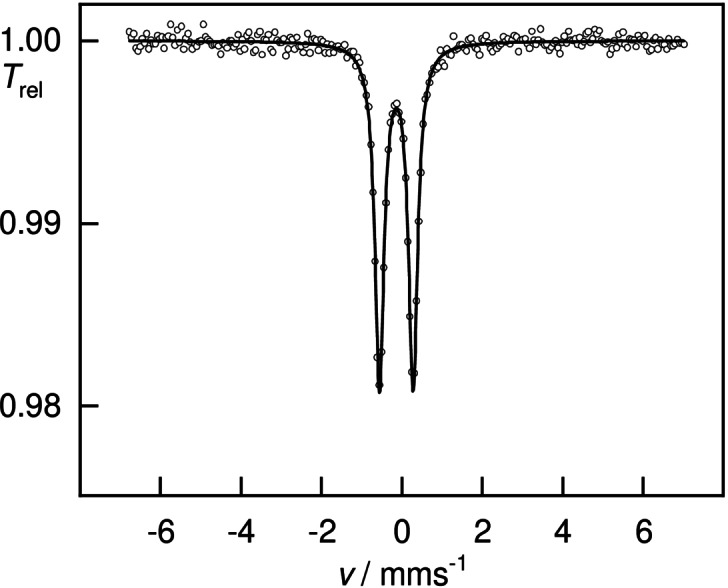
Zero‐field ^57^Fe Mössbauer spectrum of a solid sample of **4** at 77 K: doublet *δ*=−0.14(1) mm s^−1^, Δ*E*_Q_=0.84(1) mm s^−1^, *Γ*_FWHM_=0.28(1) mm s^−1^.

**Figure 7:**
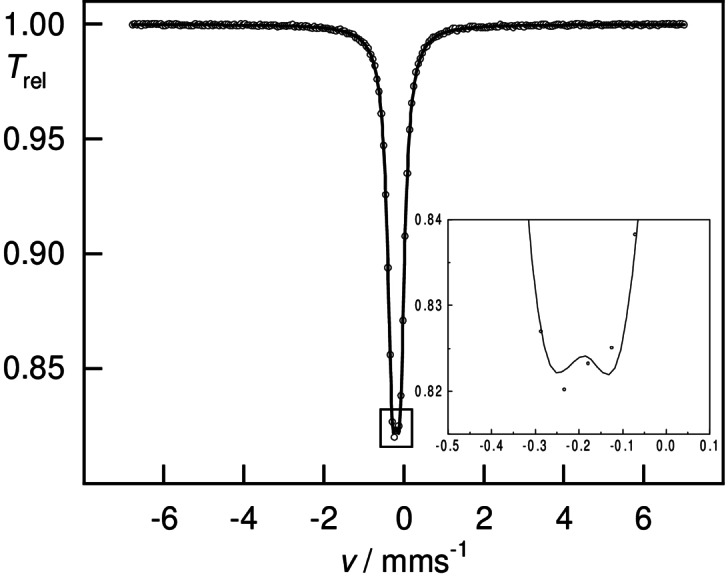
Zero‐field ^57^Fe Mössbauer spectrum of a solid sample of K_2_Fe(CO)_4_ at 77 K: doublet *δ*=−0.19(1) mm s^−1^, Δ*E*_Q_=0.19(1) mm s^−1^, *Γ*_FWHM_=0.29(1) mm s^−1^.

Prompted by its unexpected polar solubility properties and CO stretching IR bands, we aimed for further understanding of the bonding situation of **4**. Whereas the structural data are consistent with gold in the formal oxidation state of +I, the iron center presents itself electron‐rich, compared to Fe^0^(CO)_4_‐containing complexes, such as I^Mes^−Fe(CO)_4_. Zero‐field ^57^Fe Mössbauer spectroscopy was performed to assess the complex's electronic structure, (Figure 6 and Figure 7). Specifically, the Mössbauer isomer shift, *δ*, reflects the total *s*‐orbital electron density at the nucleus, which is determined by the oxidation state, the coordination geometry, spin state, and, consequently, the metal‐ligand distance as well as the degree of covalency.^[32]^ The starting material K_2_Fe(CO)_4_, which is commonly assigned a formal oxidation state of −II, was re‐investigated under the exact same conditions, for comparison.^[33]^

Intriguingly, K_2_Fe(CO)_4_ (*δ*=−0.19(1) mm s^−1^) and **4** (*δ*=−0.14(1) mm s^−1^) feature rather similar isomer shifts, which is indicative of similar electronic structures and, thus, physical oxidation states. The computational simulation of the ^57^Fe Mössbauer isomer shift at the DKH2‐TPSSh/def2‐TZVPP (Fe: CP(PPP)) level of theory matches the experimental finding almost perfectly, with a calculated isomer shift *δ* of −0.16 mm s^−1^ for **4**. The isomer shift can be rationalized by the removal of *d*‐electron density from the iron center by the four CO ligands, resulting in a less‐shielded positive charge and concentrated *s*‐electron density at the iron nuclei.^[34]^ In this regard, experimental characteristics suggest **4** to be best described as zwitterionic L^2^ClSi^+^→Au^+^←Fe^2−^(CO)_4_ (L^2^=bis‐NHI).^[35]^

**Figure 8:**
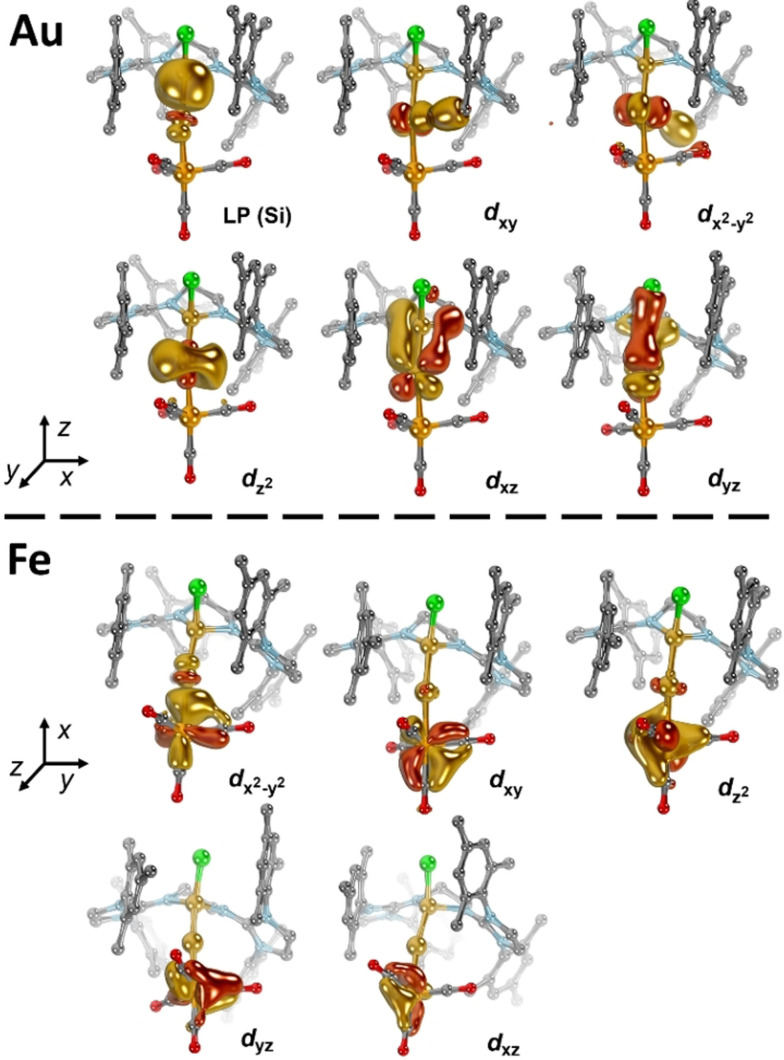
IBOs of **4** (PBE0‐D3/def2‐TZVPP//PBE0‐D3BJ/def2‐SVP) with strong valence *d*‐orbital admixture.

Electronic structure analysis was carried out using Intrinsic Bond Orbitals (IBOs^[36]^) obtained by the BP86 (Figure S37) and PBE0 (Figure 8) functionals to further pinpoint the *d*‐orbital population of the gold and iron atoms in **4**. Both methods give consistent results corresponding to formally *d* ^10^‐configured gold(+I) and iron(−II) ions. A dative interaction between the silicon lone pair (LP) and the gold metal is found with the weight of silicon amounting to 0.70 (Au: 0.30). This value, which is in the typical range of late transition metal NHC complexes, indicates a coordinative ligand‐metal interaction of considerable covalency.^[37]^ The IBOs relating to the gold *d*(xy) and *d*(x^2^‐y^2^) orbitals suggest orbital overlap with the methyl groups of the congesting mesityl substituents. The non‐bonding Au *d*(z^2^) orbital shows large admixture of the 6*s* orbital, whereas the Au *d*(xz) and *d*(yz) orbitals indicate strong π‐backbonding with the silyliumylidene ligand. This hyperconjugative interaction, which is also reflected by a calculated Löwdin bond order of 1.56, is analogous to π‐backbonding of isoelectronic phosphine ligands commonly applied in homogeneous transition metal catalysis. Note that considerable research effort has been directed recently towards engineering cationic phosphine ligands for exceedingly strong π‐backbonding.^[38]^

Also, all *d*‐orbitals of the iron ion are fully populated, which gives rise to a formal oxidation state of −II. The Fe *d*(xy), *d*(xz), and *d*(yz) orbitals engage in strong backbonding (contribution Fe≈0.75) with the CO ligands, which is in excellent agreement with the ^57^Fe Mössbauer spectroscopic data (vide supra). Further, the Fe *d*(z^2^) orbital, associated with a dative bond to the gold atom, is quite covalent (contribution Fe: 0.58; contribution Au: 0.26). Both, the Hirshfeld as well as Löwdin (Figure S39) population analysis indicate accumulation of negative partial charge at the iron atom, whereas the positive partial charge is delocalized across the silyliumylidene, gold ion, and the NHC moieties. We conclude that the computational analysis further supports a zwitterionic Si(II)^+^→Au(I)^+^←Fe(−II)^2−^ (**4**) electronic structure with considerably covalent Au−Si and, especially, Au−Fe (**4′**) bonds, as represented by the mesomeric structures **4** and **4′**.

## Conclusion

In summary, the bis‐NHI‐stabilized silyliumylidene **1** was isolated and studied for reactivity. The activation of elemental heavier chalcogens through the silyliumylidene's lone pair resulted in complexes **2 a**–**2 c**, whereas coordination to group 11 metals led to silyliumylidene‐metal complexes **3 a**–**3 c**. The first over‐all neutral heterobimetallic silyliumylidene complex **4** was isolated via nonstandard anion exchange reaction using K_2_Fe(CO)_4_. The solid‐state structure, spectroscopic analysis, and calculations of **4** reveal a rare anagostic interaction of the gold ion with the mesityl ligand, as well as strong donor and considerable π‐backbonding capabilities of the silyliumylidene ligand **1**. Computational analysis and ^57^Fe Mössbauer spectroscopy indicate **4** to feature a zwitterionic L^2^ClSi^+^→Au^+^←Fe^2−^(CO)_4_ electronic structure. Investigations on bond activation reactions and catalytic applications of **1**, **3** and **4** are currently ongoing in our group.

## Conflict of interest

The authors declare no conflict of interest.

## Supporting information

As a service to our authors and readers, this journal provides supporting information supplied by the authors. Such materials are peer reviewed and may be re‐organized for online delivery, but are not copy‐edited or typeset. Technical support issues arising from supporting information (other than missing files) should be addressed to the authors.

Supporting Information
