# Supplementary material for: A Zwitterionic Heterobimetallic Gold–Iron Complex Supported by Bis(N‐Heterocyclic Imine)Silyliumylidene
Source: Angew Chem Int Ed Engl. 2021 Sep 22;60(43):23274–80. doi: 10.1002/anie.202108146 (PMC8596601; doi:10.1002/anie.202108146)
Supplement: Supplementary file 1 — Supporting Information [file ANIE-60-23274-s001.pdf]

Angewandte Chemie
Eine Zeitschrift der Gesellschaft Deutscher Chemiker

Supporting Information

A Zwitterionic Heterobimetallic Gold–Iron Complex Supported by Bis(*N*-Heterocyclic Imine)Silyliumylidene

*Franziska Hanusch, Dominik Munz, Jörg Sutter, Karsten Meyer, and Shigeyoshi Inoue**

anie_202108146_sm_miscellaneous_information.pdf

Contents

1. Experimental Details	2
2. Crystallographic Details	29
3. Computational Details	33
4. Supplementary References	42

1. Experimental Details

General considerations: All experiments and manipulations were carried out under dry oxygen-free argon using standard Schlenk techniques and glassware or in a MBraun glovebox workstation containing an atmosphere of purified argon if not stated otherwise. Solvents were dried by standard methods (e.g. withdrawal from MBraun Solvent Purification System, storage over molecular sieves (3 Å), degassing via freeze-pump-thaw cycling, distillation from sodium/ketylradical or CaH_2). Reagents were purchased from commercial suppliers and processed as received if not stated otherwise. The starting materials IDipp-$SiCl_2$, Bis-NHI^{Mes}, and $K_2Fe(CO)_4$ were prepared according to literature procedures.[S1-4] ^{1}H-, ^{29}Si-, ^{77}Se-, ^{125}Te-, and ^{13}C{^{1}H} NMR spectra were recorded on Bruker Avance 300 MHz, 400 MHz, or 500 MHz spectrometers and referenced to residual solvent signals as internal standards (^{1}H and ^{13}C). δ(^{29}Si) was referenced to the signal of tetramethylsilane (TMS) (δ = 0 ppm) as external standard. Values for the chemical shift (*δ*) are given in parts per million. Elemental analyses were carried out by the microanalytical laboratory of the Catalysis Research Center, Technische Universität München, using a HEKAtech EURO EA instrument equipped with a CHNS combustion analyze. Melting Points (M.P.) were determined in sealed glass capillaries under inert gas by a Büchi M-565 melting point apparatus. ESI-MS analysis was conducted on a Bruker HCT Instrument with a dry gas temperature of 300 °C and an injection speed of 240 µLs-1. Samples were prepared in a glovebox and spectra were visualized using OriginPro 2018. Zero-field ^{57}Fe-Mößbauer spectra were recorded on a WissEl Mößbauer spectrometer (MRG-500) at 77 K in constant acceleration mode. ^{57}Co/Rh was used as the radiation source. Least-square fitting of the Lorentzian signals was carried out with the Mfir software, developed by Dr. Eckhard Bill (MPI Mülheim/Ruhr). The minimum experimental line widths were 0.21 mms^{-1}. The temperature of the samples was controlled by an MBBC-HE0106 MÖSSBAUER He/N_2 cryostat within an accuracy of ±0.3 K. Isomer shifts were determined relative to α-iron at 298 K.

Abbreviations: s = singlet, d = doublet, quint = quintet, t = triplet, sept = septet, br = broad, n.a. = not applicable/no answer, n.r. = not resolved, n.o. = not observed.

Procedure for the isolation of [Bis-NHI^{Mes}-SiCl]Cl (1)

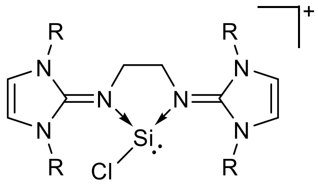

1[Cl⁻]

70 ml of toluene was added to a mixture of Bis-NHI^{Mes} (750 mg, 1.13 mmol) and IDipp-$SiCl_2$ (605 mg, 1.24 mmol, 1.1 eq) in one portion at room temperature. After filtration of very little insoluble residue, the mixture was stirred for 16 hours. The product was separated from the solution by filtration and consequently washed with 20 ml of toluene. The colorless, pearly solid was dried in fine vacuum to obtain 654 mg (76%) of **1** as a colorless solid. X-ray quality crystals of **1** were obtained by gas phase diffusion of solvent between a solution of **1** in *ortho*-difluorobenzene and a reservoir of diethyl ether over 2 days.

^{1}H NMR (500.1 MHz, CD_3CN): *δ* [ppm] = 6.97 (s, 8H, Mes*H*-3,5), 6.80 (s, 4H, NC*H*), 2.93 (br, 2H C_2H_4), 2.61 (br, 2H, C_2H_4), 2.33 (s, 12H, Mes-*p*-C*H*$_3$), 2.01 (s, 24H, Mes-*o*-C*H*$_3$).

^{13}C{^{1}H} NMR (125.8 MHz, CD_3CN): *δ* [ppm] = 147.90 (N*C*N), 141.61 (Mes*C*-1), 137.16 (Mes*C*-2,6), 132.61 (Mes*C*-4), 130.19 (Mes*C*-3,5) 119.83 (N*C*H), 47.26 (C_2H_4), 21.24 (p-Mes*C*H$_3$), 18.32 (o-Mes*C*H$_3$).

^{29}Si NMR (99.4 MHz, CD_3CN): *δ* [ppm] = 1.54.

Elemental analysis: [763.93] calcd: C 69.18, H 6.86, N 11.00; found: C 65.73, H 6.71, N 9.77. (Consistently low C values can be explained by the formation of silicon carbide.)

M.P.: 215.4 °C (decomposition, color change to dark red)

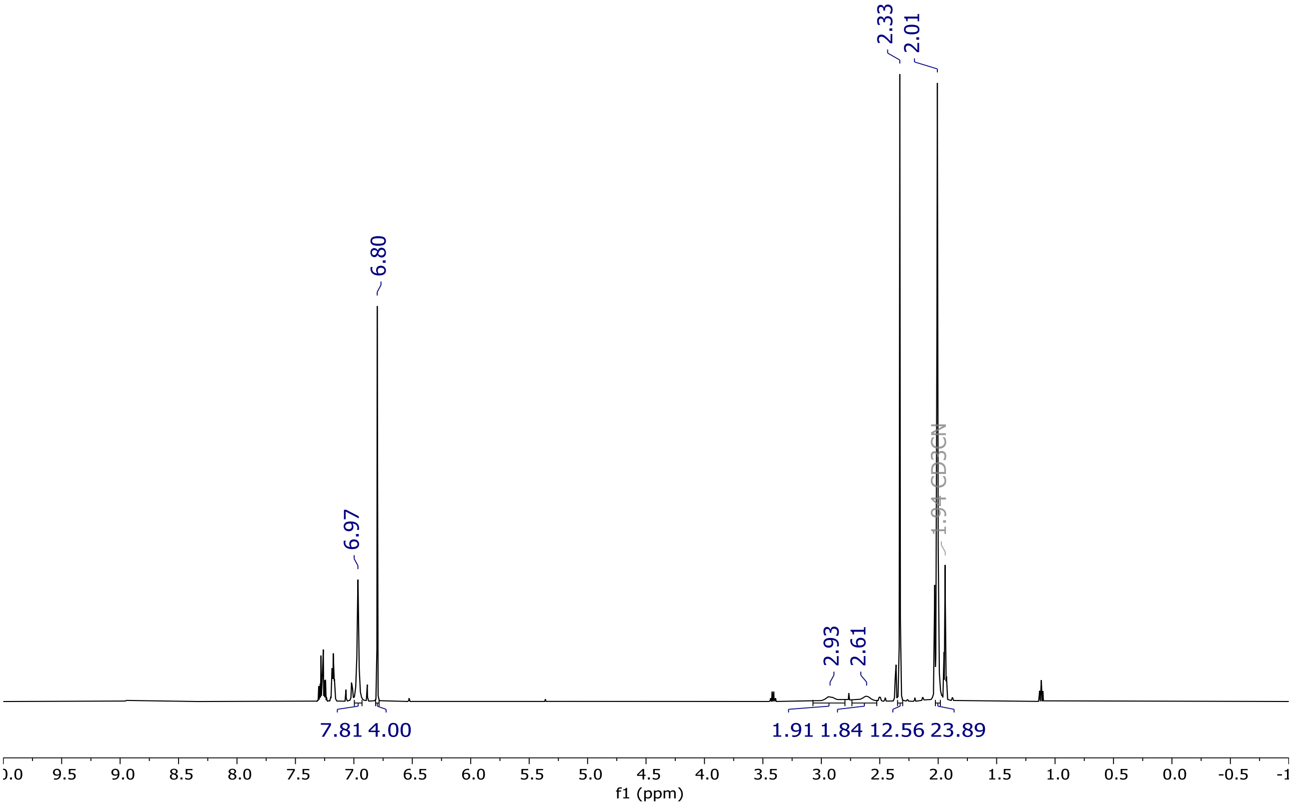

Figure S1 ^{1}H NMR spectrum of compound **1** in CD_3CN at 300 K. Trace amounts of *o*-difluorobenzene, toluene and diethylether are contained.

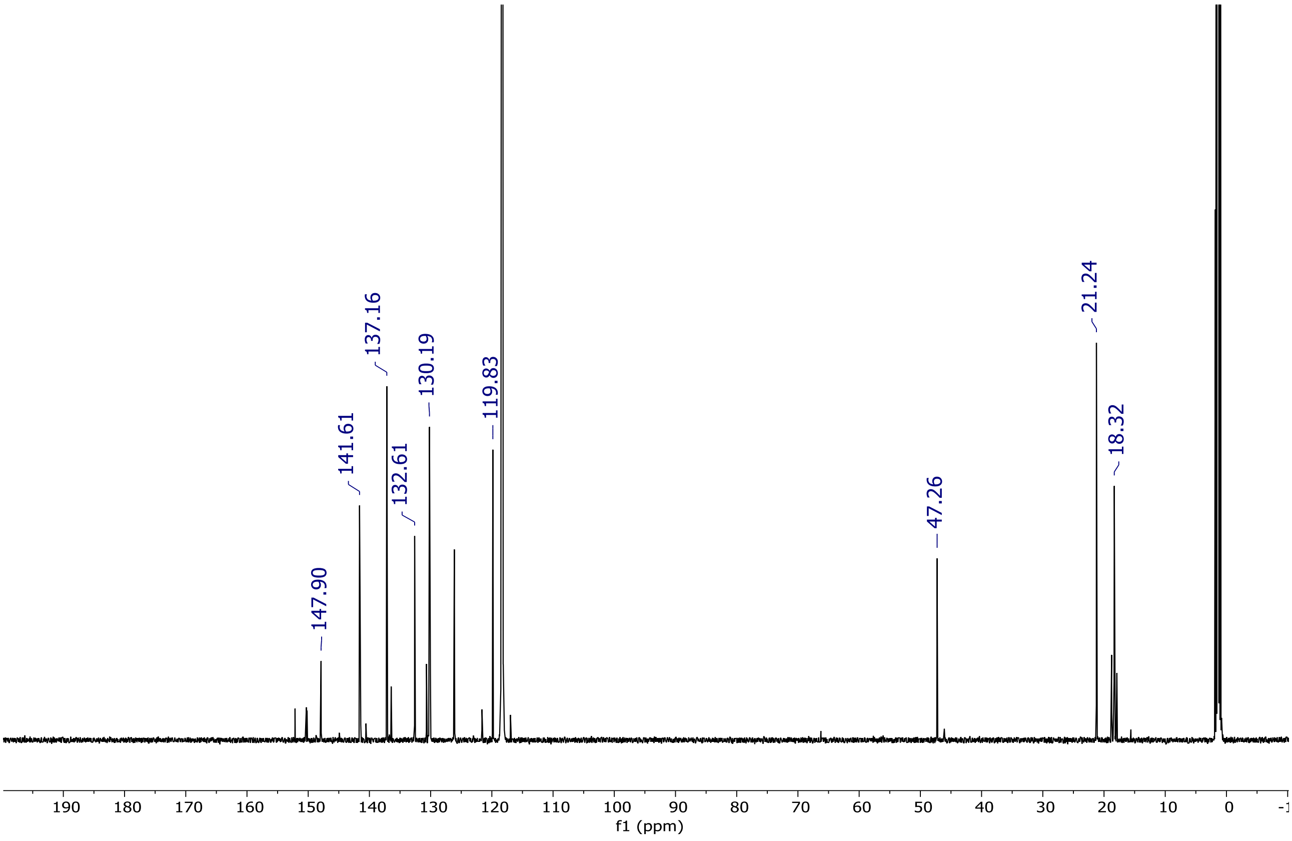

Figure S2 $^{13}C\{H\}$ NMR spectrum of compound **1** in CD_3CN at 300 K. Trace amounts of *o*-difluorobenzene and toluene are contained.

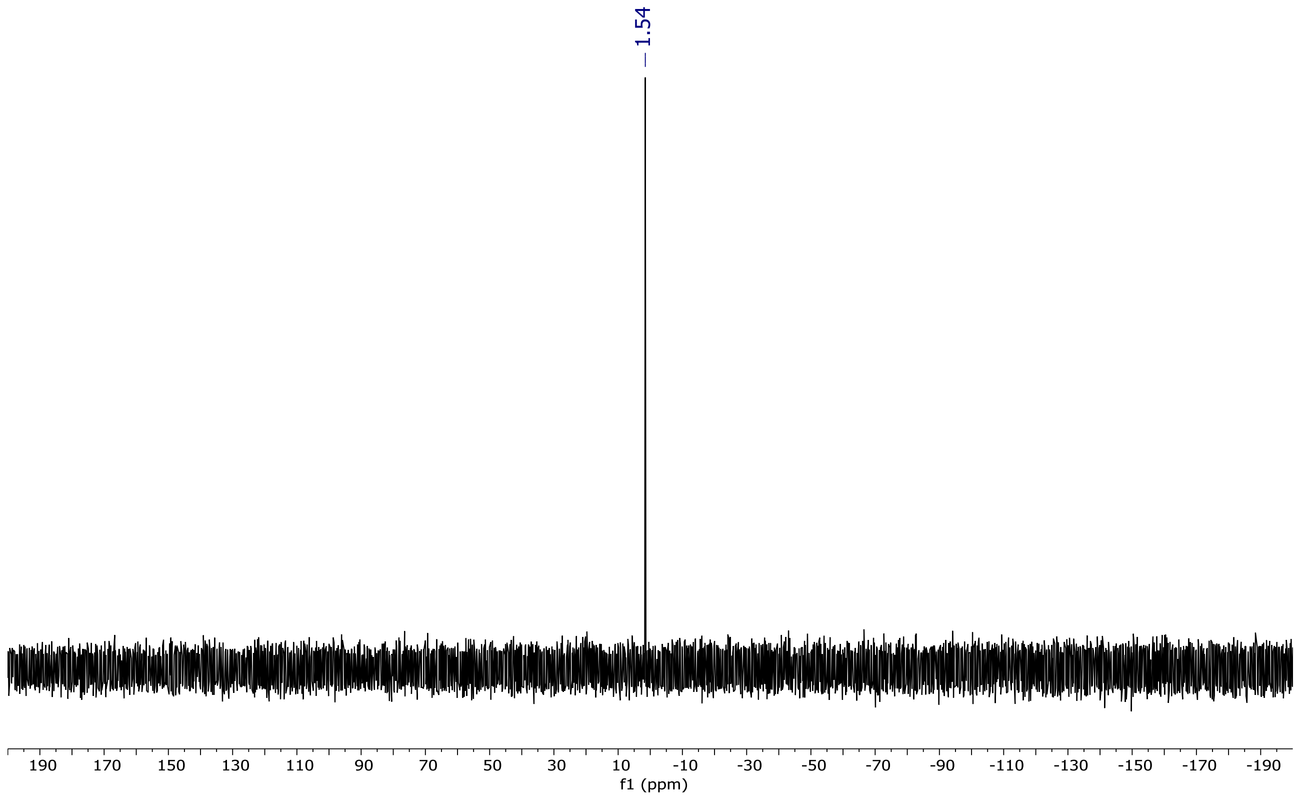

Figure S3 ^{29}Si NMR spectrum of compound **1** in CD_3CN at 300 K.

Procedure for the isolation of [Bis-NHI^{Mes}-SiClS]Cl (2a)

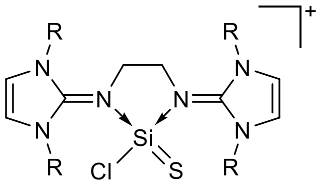

2a[Cl$^-$]

3 ml of acetonitrile was added to a mixture of **1** (80 mg, 0.10 mmol) and S_8 (6.7 mg, 0.21 mmol, 2 eq.) in one portion at room temperature and the mixture was stirred for 45 min. Excess of sulfur was separated by filtration and the solution was evaporated under reduced pressure to yield the crude product as an off-white solid. The product was washed with 0.5 ml of toluene and dried in fine vacuum to obtain 80.0 mg (96%) of **2a** as a colorless solid. X-ray quality crystals were obtained by gas phase diffusion of solvent between a solution of **2a** in acetonitrile and a reservoir of THF over 3 weeks.

^{1}**H NMR** (500.1 MHz, CD_3CN): δ [ppm] = 7.04 (s, 4H, NC*H*), 7.02 (s, 4H, Mes*H*-3,5), 6.93 (s, 4H, Mes*H*-3,5), 3.13 (m, 2H C_2H_4), 2.78 (m, 2H, C_2H_4), 2.36 (s, 12H, Mes-C*H*$_3$), 2.23 (s, 12H, Mes-C*H*$_3$), 2.03 (s, 12H, Mes-C*H*$_3$).
^{13}**C{^{1}H} NMR** (125.8 MHz, CD_3CN): δ [ppm] = 146.70 (N*C*N), 141.68 (Mes*C*-1), 136.62 (Mes*C*-2,6), 136.41 (Mes*C*-2,6), 132.32 (Mes*C*-4), 130.89 (Mes*C*-3,5), 130.55 (Mes*C*-3,5), 122.14 (N*C*H), 47.59 (*C*$_2$H$_4$), 21.33 (p-Mes*C*H$_3$), 18.91 (o-Mes*C*H$_3$), 18.57 (o-Mes*C*H$_3$).
^{29}**Si NMR** (99.4 MHz, CD_3CN): δ [ppm] = –26.73.
MS (ESI$^+$) *m/z* calcd: 759.34; *m/z* found: 759.3
Elemental analysis: [794.31] calcd: C 66.39, H 6.59, N 10.56, S 4.03; found: C 63.78, H 6.45, N 10.36, S 3.35.
(Consistently low C values can be explained by the formation of silicon carbide.)
M.P.: 268.2 °C (decomposition, color change to black)

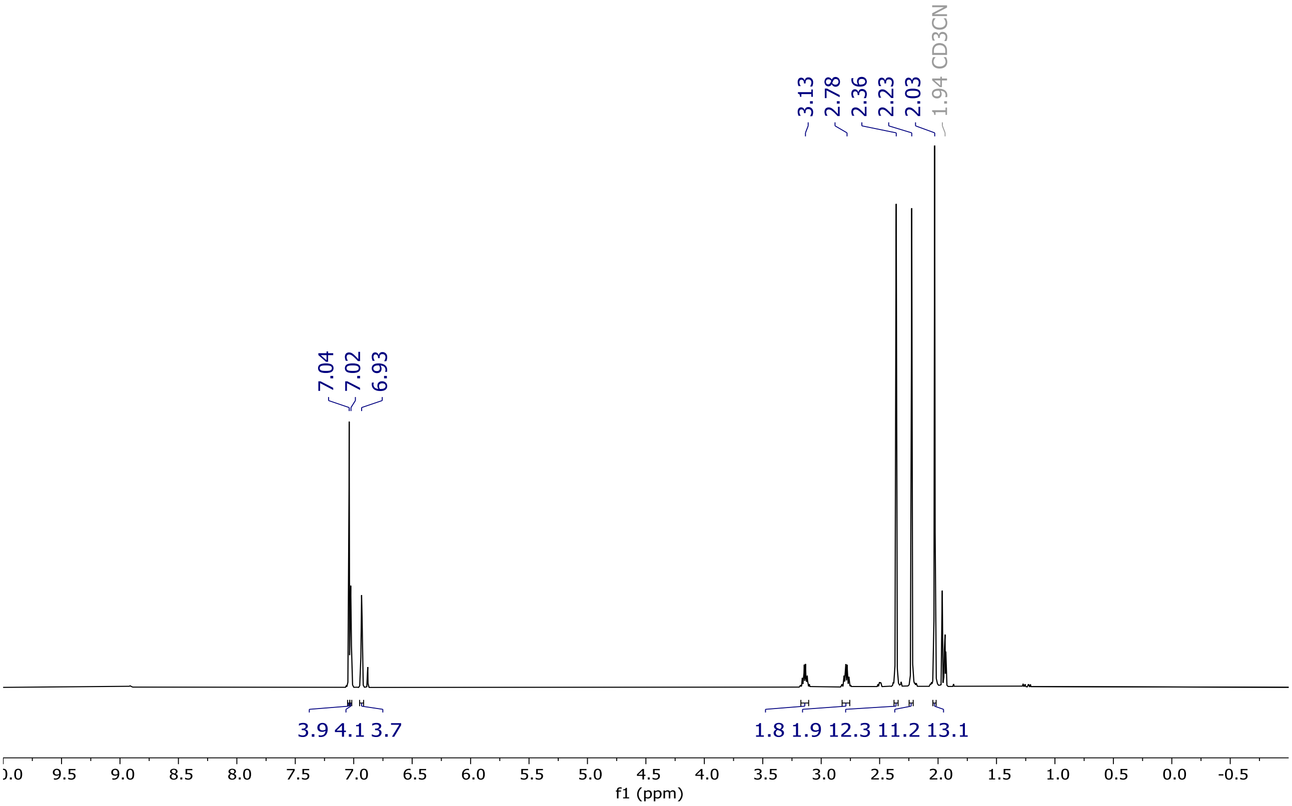

Figure S4 ^{1}H NMR spectrum of compound **2a** in CD_3CN at 300 K. Trace amounts of acetonitrile are contained.

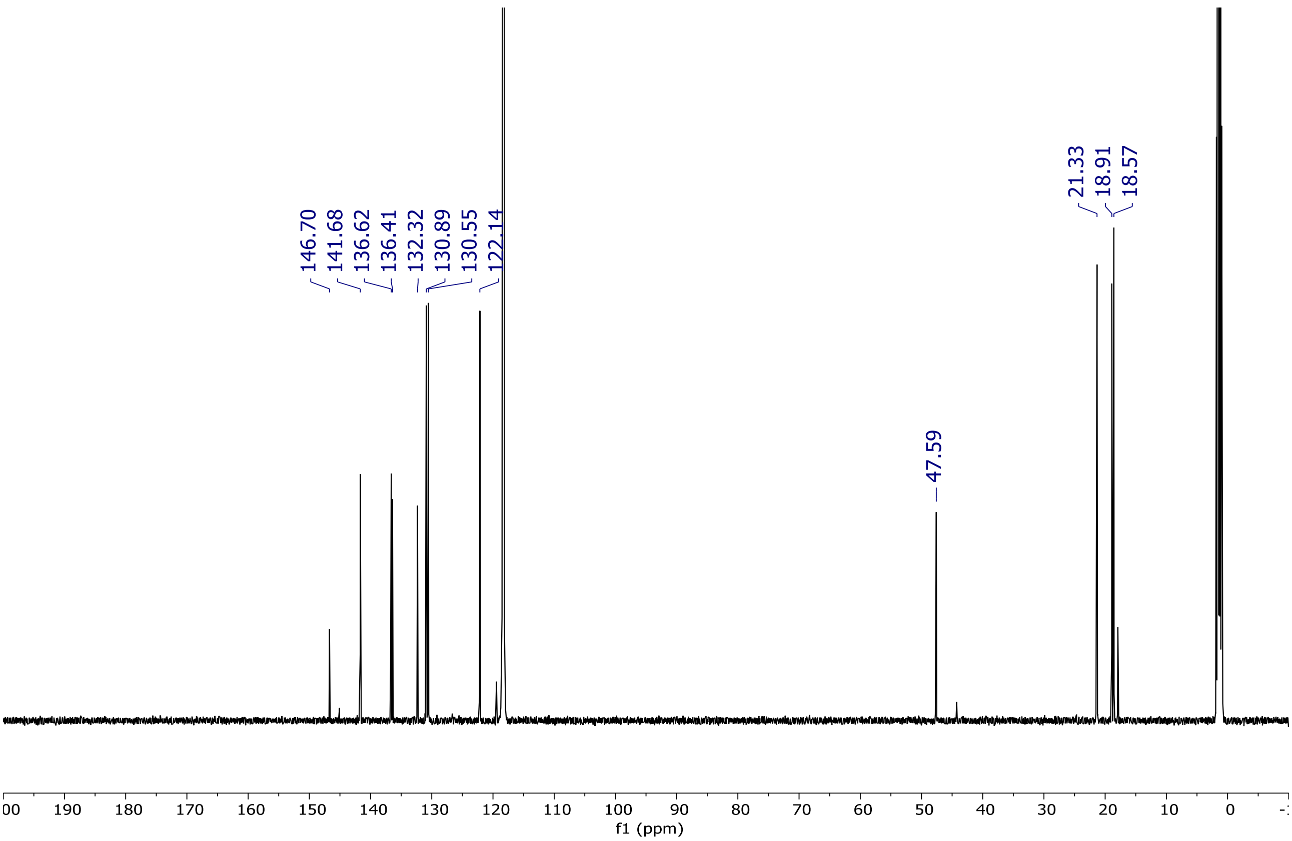

Figure S5 ^{13}C{H} NMR spectrum of compound **2a** in CD_3CN at 300 K.

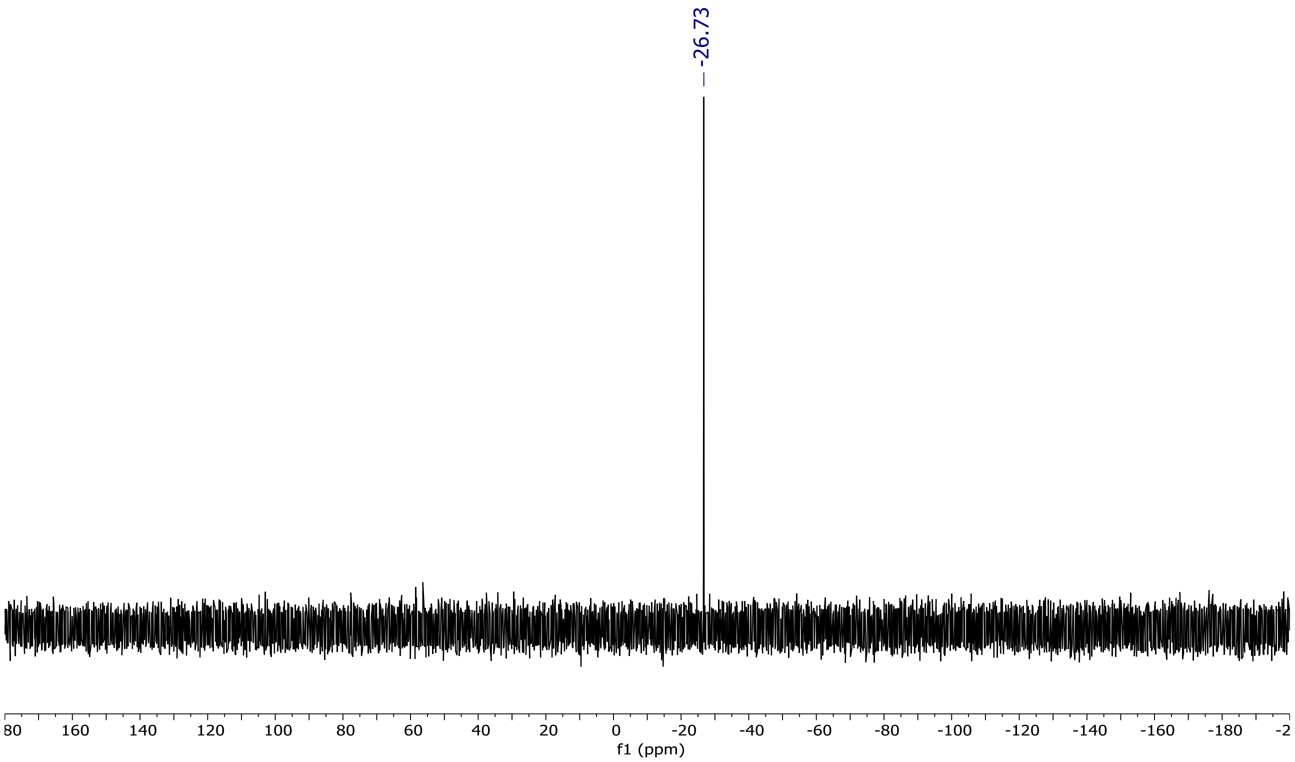

Figure S6 ^{29}Si NMR spectrum of compound **2a** in CD_3CN at 300 K.

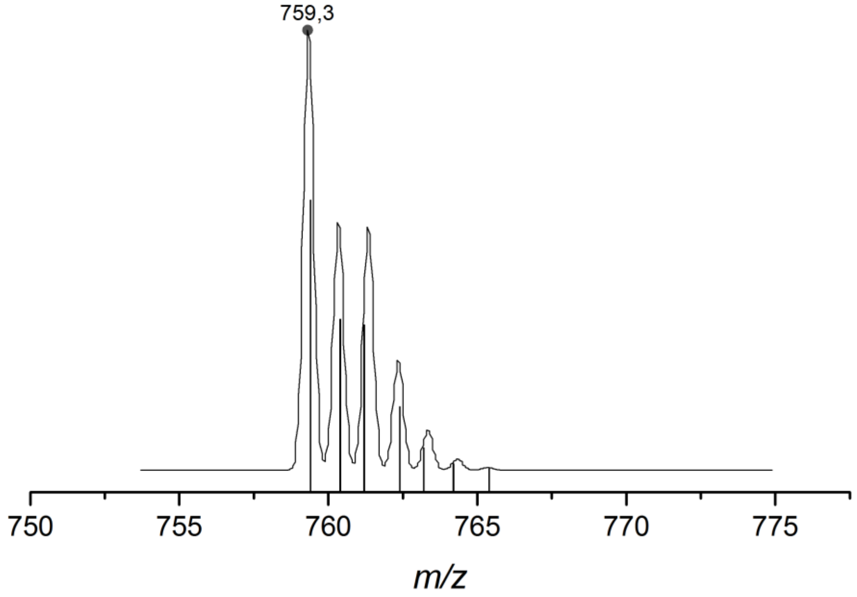

Figure S7. ESI-MS spectrum (detail view) of **2a** (positive mode, 300 °C, –3500 V; line: measured spectrum; bars: simulated spectrum).

Procedure for the isolation of [Bis-NHI^{Mes}-SiClSe]Cl (2b)

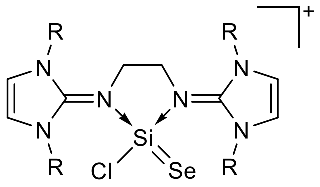

2b[Cl$^-$]

3 ml of acetonitrile was added to a mixture of **1** (80 mg, 0.10 mmol) and Se (16.5 mg, 0.21 mmol, 2 eq.) in one portion at room temperature and the mixture was stirred for 6 h. Excess of selenium was separated by filtration and the solution was evaporated under reduced pressure to yield the crude product as a slightly yellow solid. The product was washed with 0.5 ml of toluene and dried in fine vacuum to obtain 82.0 mg (93%) of **2b** as a colorless solid.

^{1}H NMR (500.1 MHz, CD_3CN): *δ* [ppm] = 7.07 (s, 4H, NC*H*), 7.02 (s, 4H, Mes*H*-3,5), 6.93 (s, 4H, Mes*H*-3,5), 3.16 (m, 2H C_2H_4), 2.77 (m, 2H, C_2H_4), 2.35 (s, 12H, Mes-CH_3), 2.25 (s, 12H, Mes-CH_3), 2.05 (s, 12H, Mes-CH_3).

^{13}C{^{1}H} NMR (125.8 MHz, CD_3CN): *δ* [ppm] = 146.63 (N*C*N), 141.68 (Mes*C*-1), 136.57 (Mes*C*-2,6), 136.37 (Mes*C*-2,6), 132.37 (Mes*C*-4), 130.93 (Mes*C*-3,5), 130.57 (Mes*C*-3,5), 122.26 (N*C*H), 48.04 (C_2H_4), 21.34 (p-Mes*C*H_3), 19.24 (o-Mes*C*H_3), 18.93 (o-Mes*C*H_3).

^{29}Si NMR (99.4 MHz, CD_3CN): *δ* [ppm] = –30.99.

^{77}Se NMR (76.3 MHz, CD_3CN): *δ* [ppm] = –388.78.

MS (ESI$^+$) *m/z* calcd: 807.29; *m/z* found: 807.4

Elemental analysis: [842.26] calcd: C 62.70, H 6.22, N 9.97; found: C 61.41, H 6.05, N 9.47. (Consistently low C values can be explained by the formation of silicon carbide.)

M.P.: 254.5 °C (decomposition, color change to green-black)

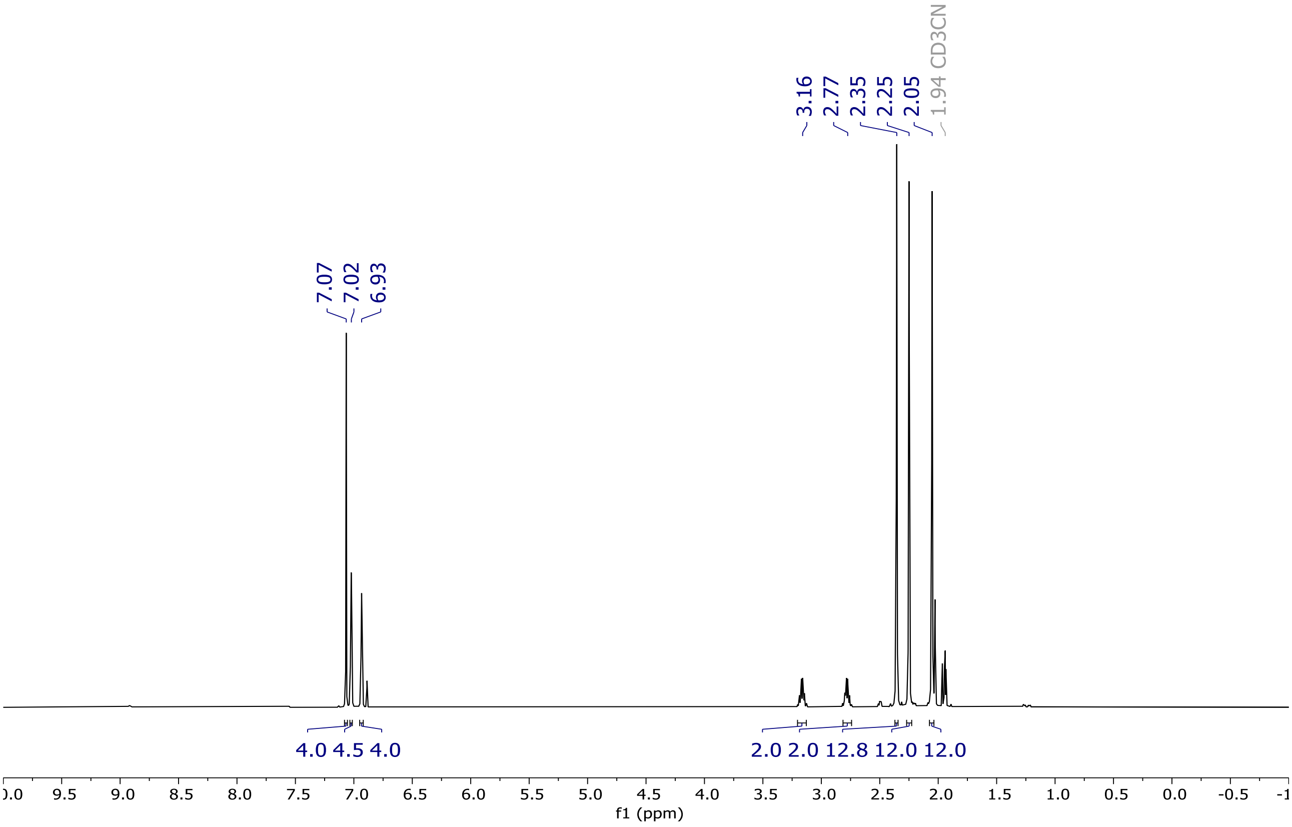

Figure S8 ^{1}H NMR spectrum of compound **2b** in CD_3CN at 300 K. Trace amounts of acetonitrile are contained.

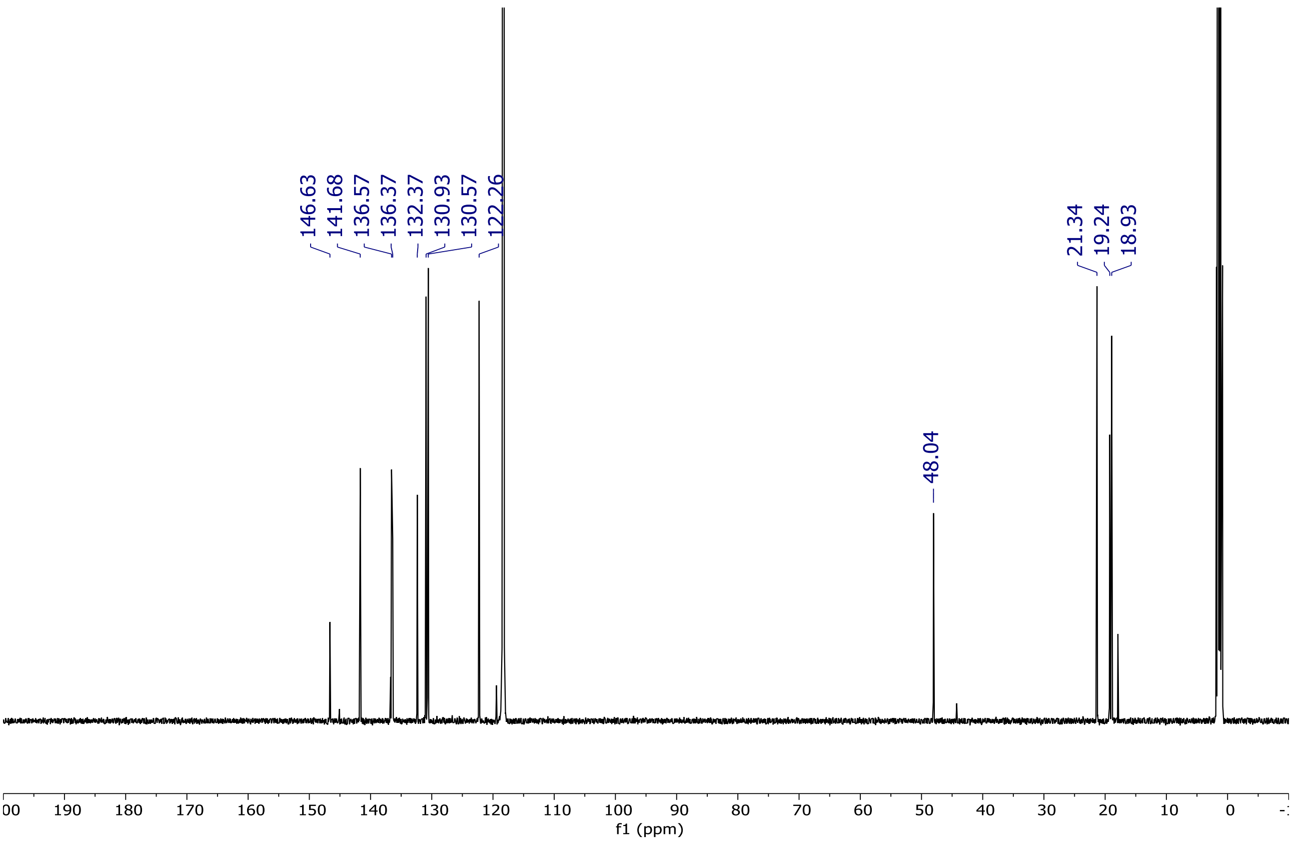

Figure S9 ^{13}C{H} NMR spectrum of compound **2b** in CD_3CN at 300 K.

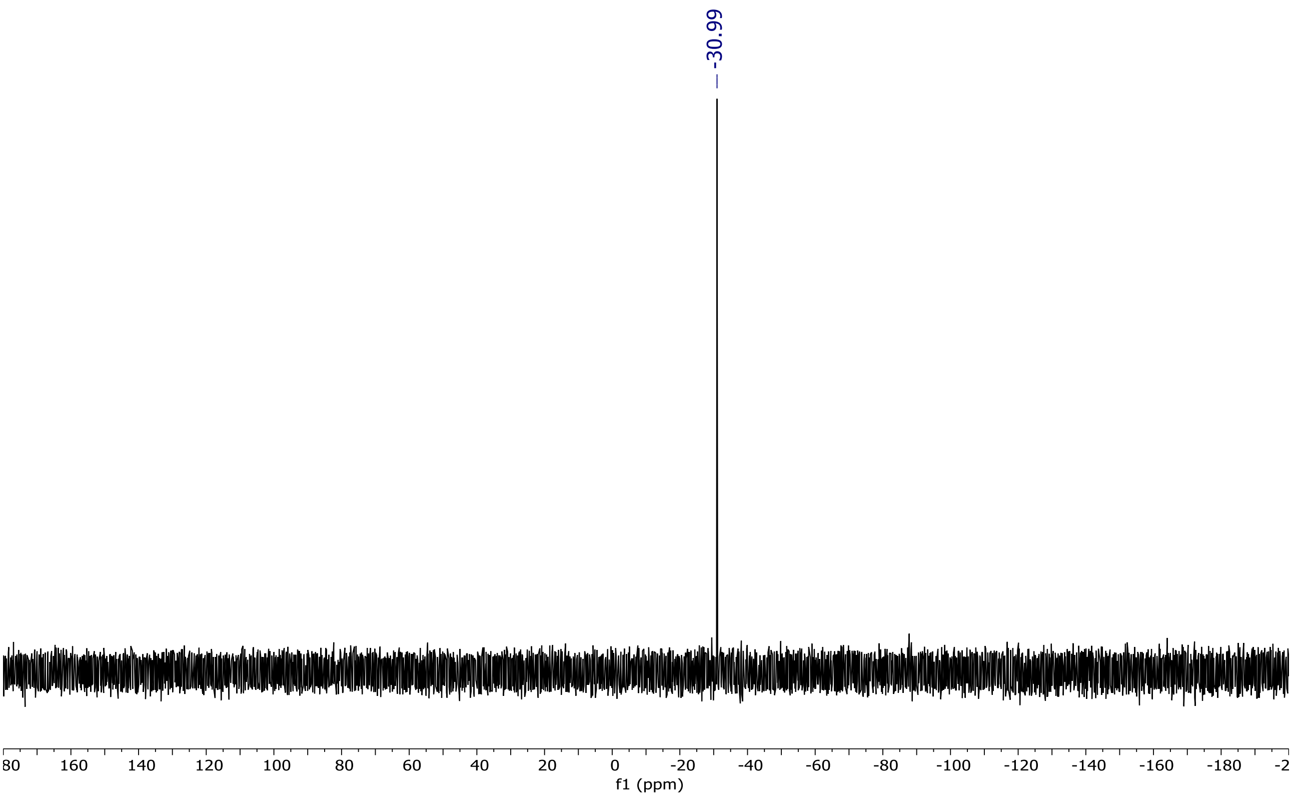

Figure S10 ^{29}Si NMR spectrum of compound **2b** in CD_3CN at 300 K.

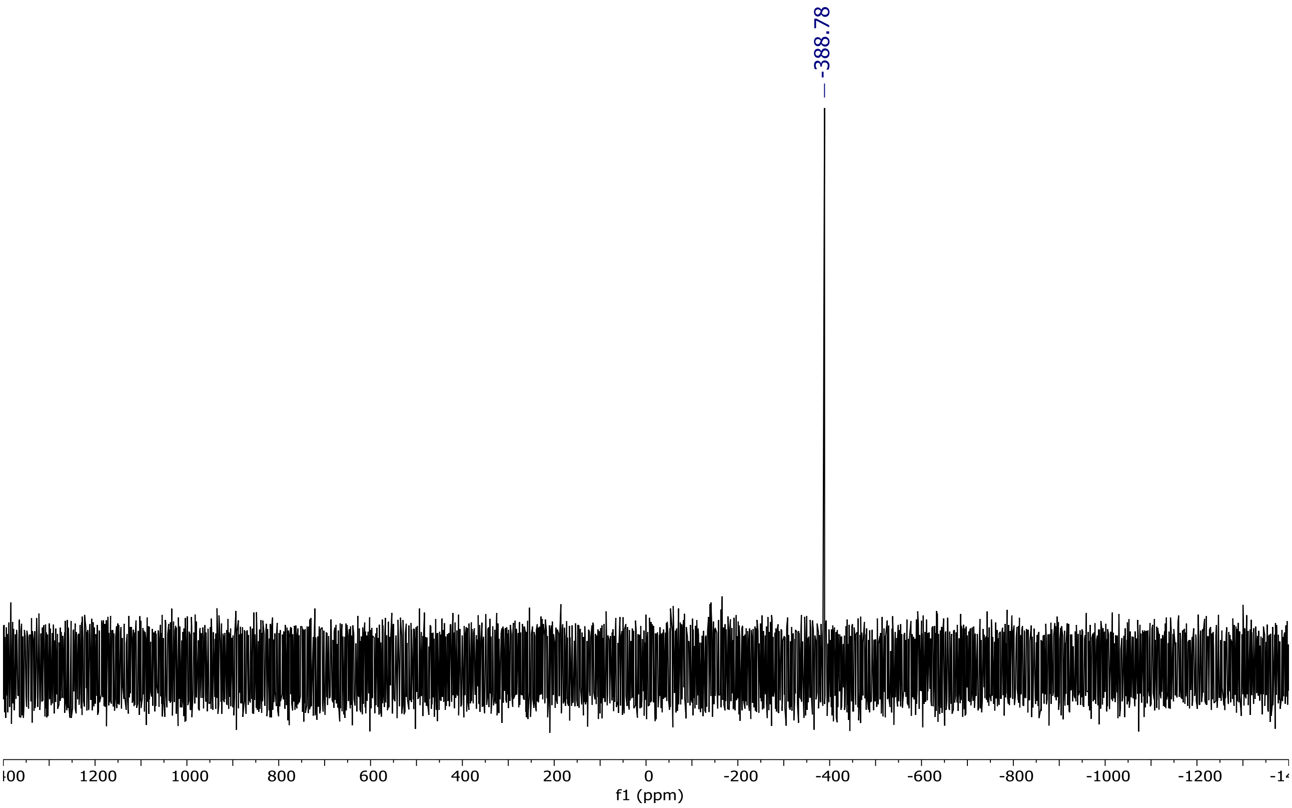

Figure S11 ^{77}Se NMR spectrum of compound **2b** in CD_3CN at 300 K.

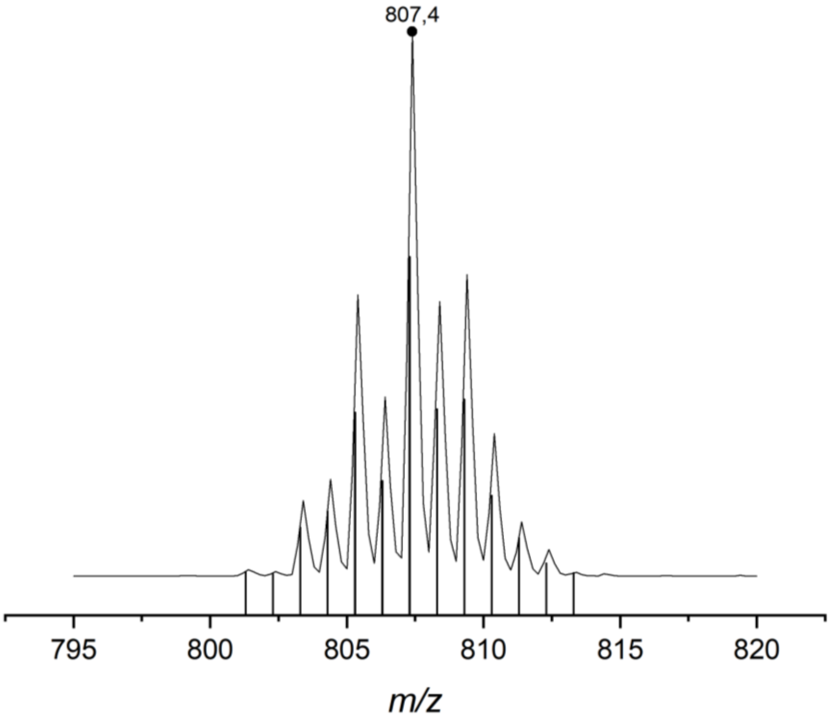

Figure S 12 ESI-MS spectrum (detail view) of **2b** (positive mode, 300 °C, –3500 V; line: measured spectrum; bars: simulated spectrum).

Procedure for the isolation of [Bis-NHI^{Mes}-SiClTe]Cl (2c)

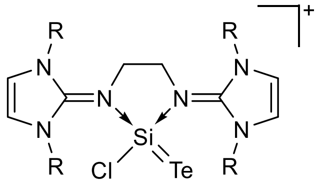

2c[Cl$^-$]

3 ml of acetonitrile was added to a mixture of **1** (80 mg, 0.10 mmol) and Te (26.7 mg, 0.21 mmol, 2 eq.) in one portion at room temperature and the mixture was stirred for 72 h. Excess of tellurium was separated by filtration and the solution was evaporated under reduced pressure to yield the crude product as a slightly yellow solid. The product was washed with 0.5 ml of toluene and dried in fine vacuum to obtain 86.0 mg (92%) of **2c** as a colorless solid.

^{1}H NMR (500.1 MHz, CD_3CN): *δ* [ppm] = 7.11 (s, 4H, NC*H*), 7.05 (s, 4H, Mes*H*-3,5), 6.97 (s, 4H, Mes*H*-3,5), 3.22 (m, 2H C_2H_4), 2.79 (m, 2H, C_2H_4), 2.38 (s, 12H, Mes-CH_3), 2.31 (s, 12H, Mes-CH_3), 2.11 (s, 12H, Mes-CH_3).

^{13}C{^{1}H} NMR (125.8 MHz, CD_3CN): *δ* [ppm] = 146.60 (N*C*N), 141.74 (Mes*C*-1), 136.47 (Mes*C*-2,6), 136.28 (Mes*C*-2,6), 132.45 (Mes*C*-4), 131.02 (Mes*C*-3,5), 130.61 (Mes*C*-3,5), 122.45 (N*C*H), 48.81 (C_2H_4), 21.34 (p-Mes*C*H_3), 20.00 (o-Mes*C*H_3), 19.74 (o-Mes*C*H_3).

^{29}Si NMR (99.4 MHz, CD_3CN): *δ* [ppm] = –59.14.

^{125}Te NMR (126.24 MHz, CD_3CN): *δ* [ppm] = –1049.36.

MS (ESI$^+$) *m/z* calcd: 857.28; *m/z* found: 857.4

Elemental analysis: [892.25] calcd: C 59.28, H 5.88, N 9.43; found: C 60.11, H 6.19, N 9.28.

M.P.: 271.8 °C (decomposition, color change to black)

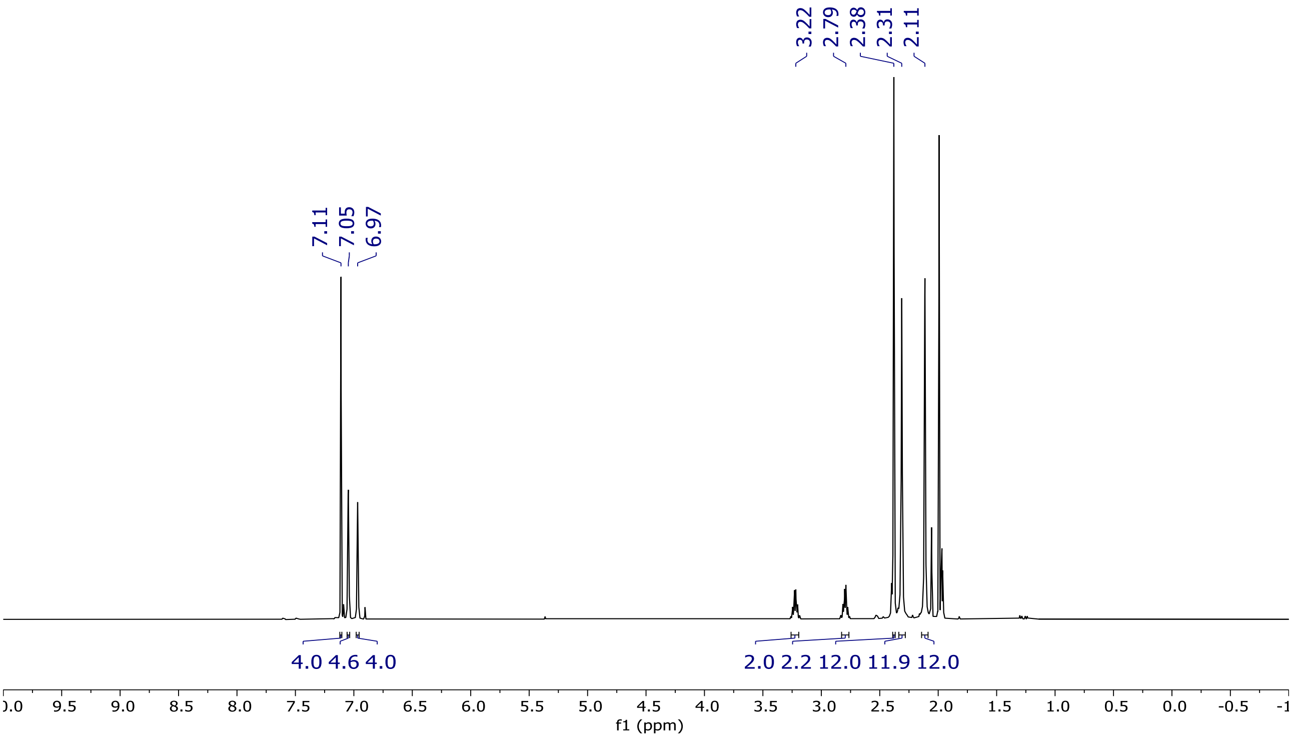

Figure S13 ^{1}H NMR spectrum of compound **2c** in CD_3CN at 300 K. Acetonitrile from synthesis is contained.

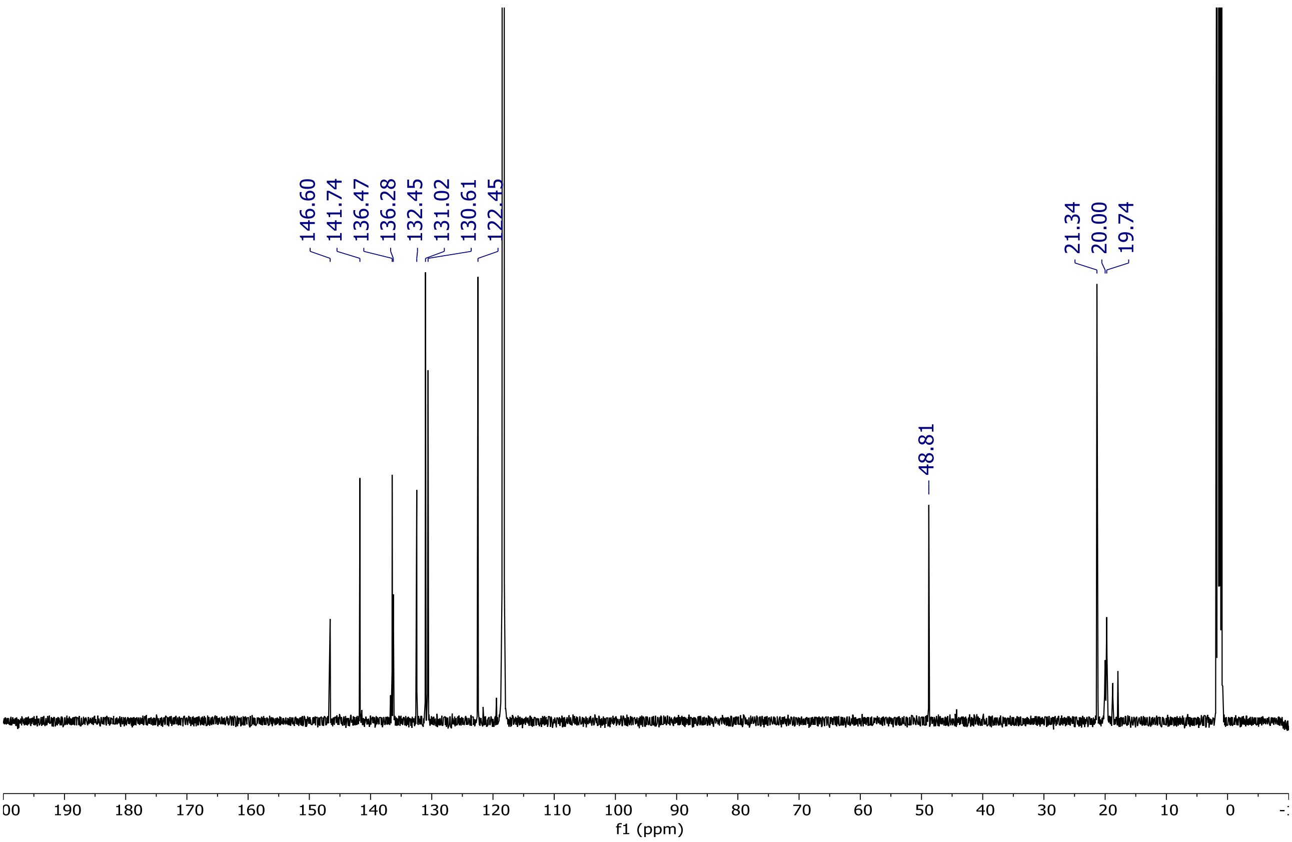

Figure S14 ^{13}C{H} NMR spectrum of compound **2c** in CD_3CN at 300 K.

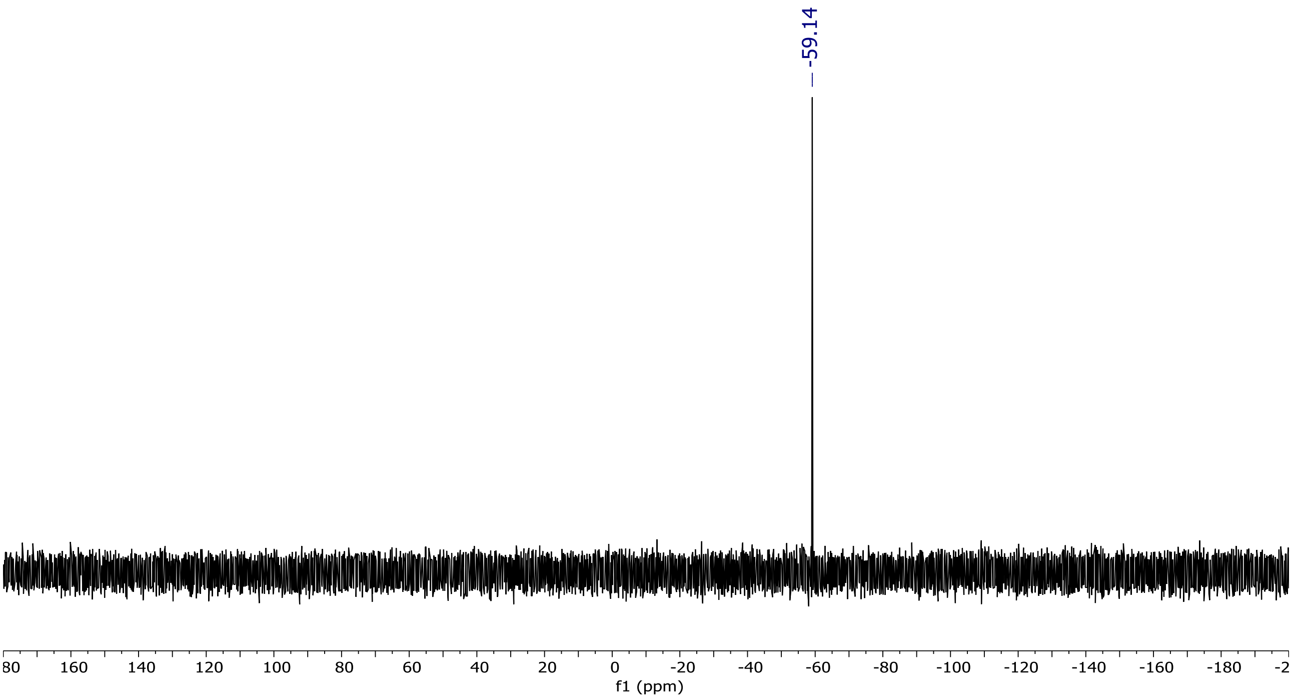

Figure S15 ^{29}Si NMR spectrum of compound **2c** in CD_3CN at 300 K.

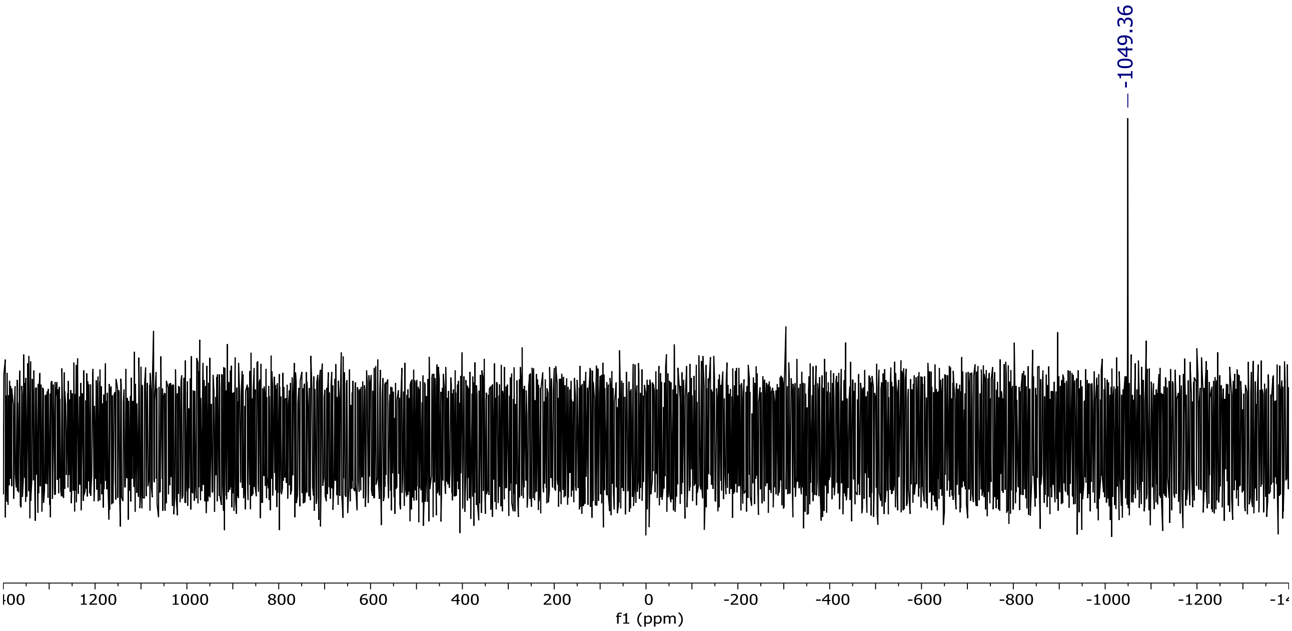

Figure S16 ^{125}Te NMR spectrum of compound **2c** in CD_3CN at 300 K.

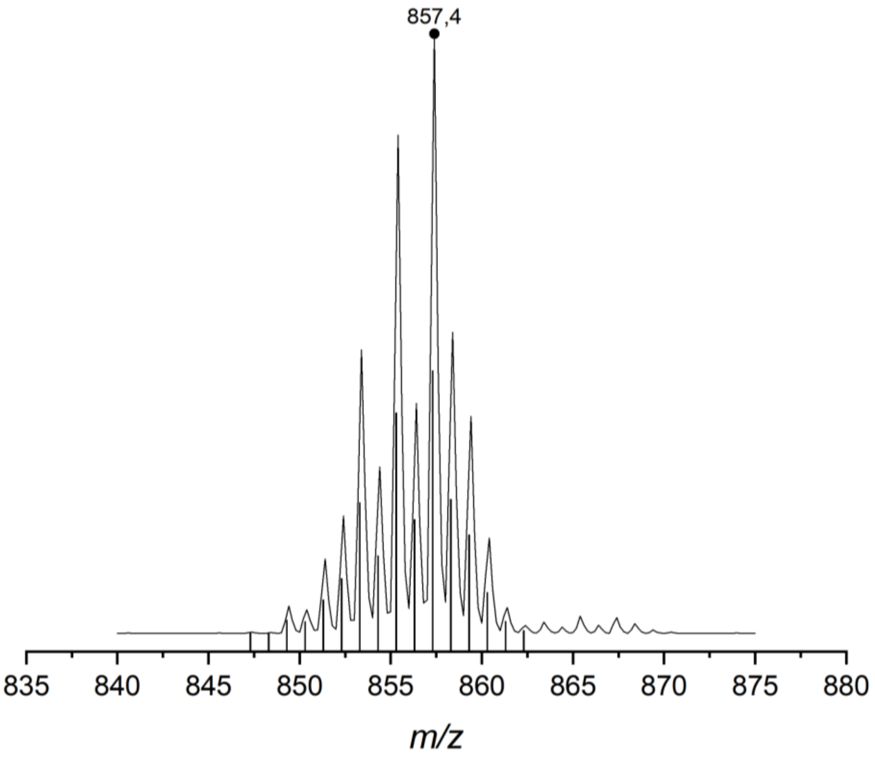

Figure S 17 ESI-MS spectrum (detail view) of **2c** (positive mode, 300 °C, –3500 V; line: measured spectrum; bars: simulated spectrum).

Procedure for the isolation of [Bis-NHI^{Mes}-SiCl(CuCl)]Cl (3a)

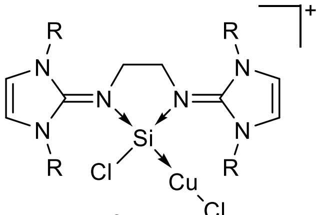

3a[Cl$^-$]

3 ml of acetonitrile was added to a mixture of **1** (80 mg, 0.10 mmol) and CuCl (10.4 mg, 0.10 mmol, 1 eq.) in one portion at room temperature and the mixture was stirred for 6 h. Minor amount of insoluble residue was separated by filtration and the solution was evaporated under reduced pressure to yield the crude product as a slightly yellow solid. The product was washed with 0.5 ml of toluene and dried in fine vacuum to obtain 89.0 mg (94%) of **3a** as a colorless solid.

^{1}H NMR (500.1 MHz, CD_3CN): *δ* [ppm] = 7.10 (s, 4H, Mes*H*-3,5), 7.07 (s, 4H, Mes*H*-3,5), 7.06 (s, 4H, NC*H*), 3.03 (m, 2H C_2H_4), 2.80 (m, 2H, C_2H_4), 2.38 (s, 12H, Mes-C*H*$_3$), 2.10 (s, 12H, Mes-C*H*$_3$), 2.01 (s, 12H, Mes-C*H*$_3$).
^{13}C{^{1}H} NMR (125.8 MHz, CD_3CN): *δ* [ppm] = 147.01 (N*C*N), 142.11 (Mes*C*-1), 136.14 (Mes*C*-2,6), 136.05 (Mes*C*-2,6), 131.86 (Mes*C*-4), 131.35 (Mes*C*-3,5), 131.00 (Mes*C*-3,5), 121.80 (N*C*H), 48.48 (C_2H_4), 21.30 (p-Mes*C*H$_3$), 18.97 (o-Mes*C*H$_3$).
^{29}Si NMR (99.4 MHz, CD_3CN): *δ* [ppm] = 13.84.
MS (ESI$^+$) *m/z* calcd: 827.47; *m/z* found: 827.4
Elemental analysis: [860.24] calcd: C 61.24, H 6.07, N 9.74; found: C 54.36, H 5.43, N 8.27.
(Consistently low C values can be explained by the formation of silicon carbide.)
M.P.: 168.9 °C (decomposition, color change to black)

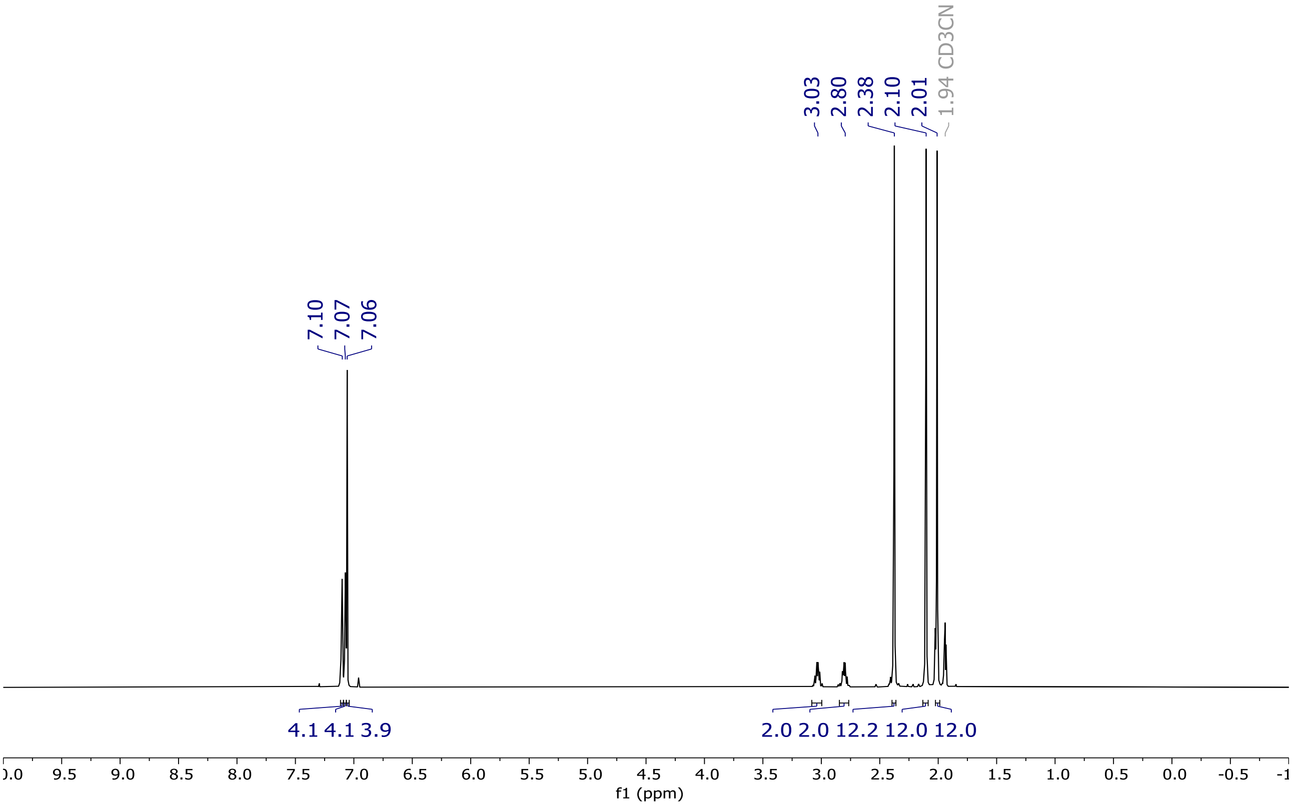

Figure S18 ^{1}H NMR spectrum of compound **3a** in CD_3CN at 300 K.

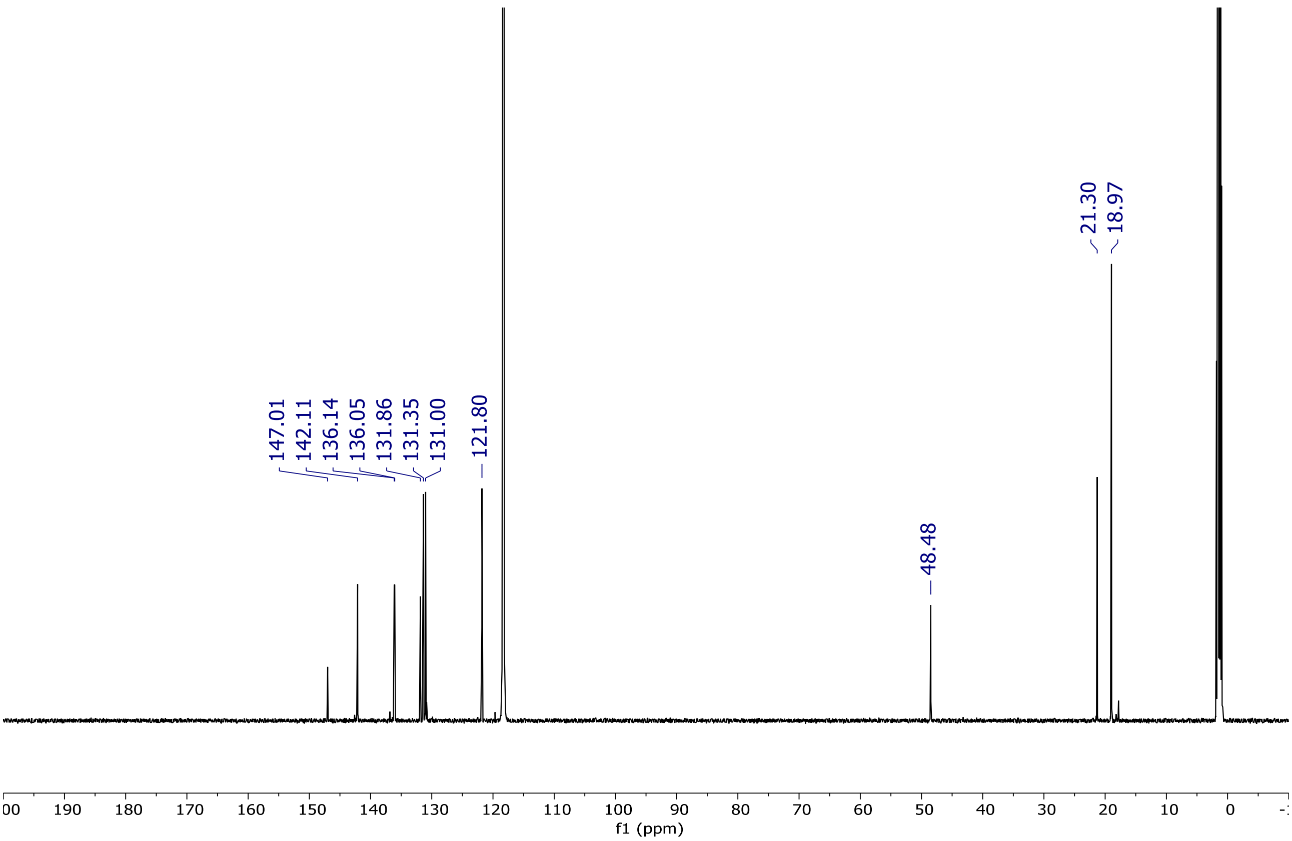

Figure S19 ^{13}C{H} NMR spectrum of compound **3a** in CD_3CN at 300 K.

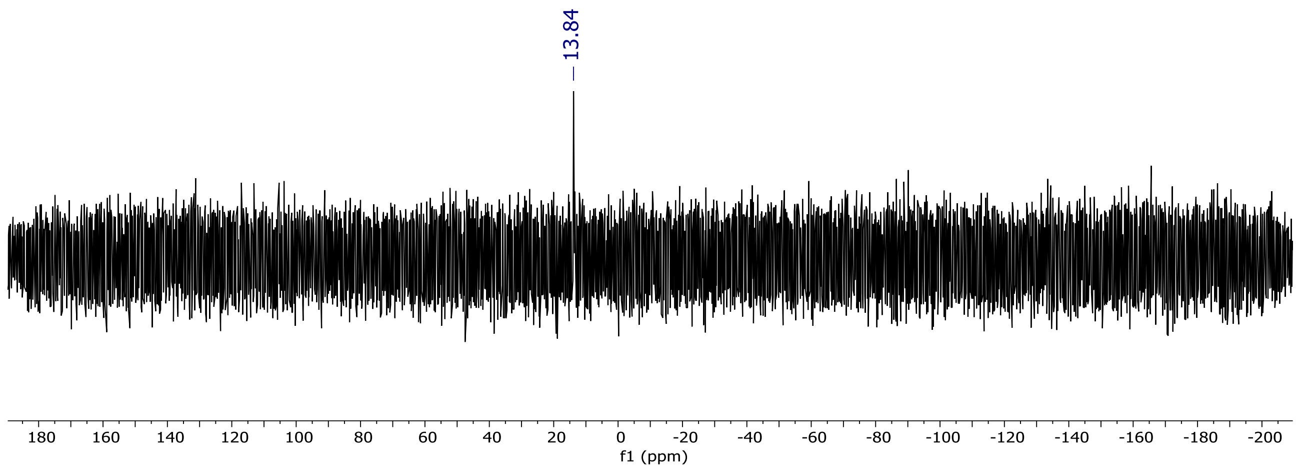

Figure S20 ^{29}Si NMR spectrum of compound **3a** in CD_3CN at 300 K.

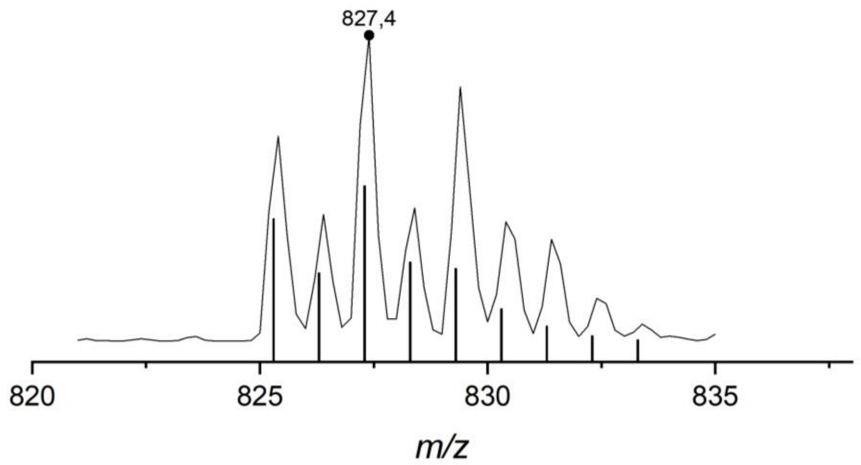

Figure S 21 ESI-MS spectrum (detail view) of **3a** (positive mode, 300 °C, –2500 V; line: measured spectrum; bars: simulated spectrum).

Procedure for the isolation of [Bis-NHI^{Mes}-SiCl(AgCl)]Cl (3b)

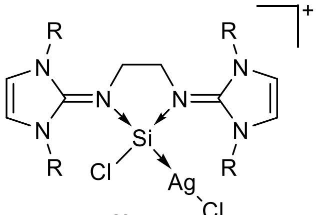

3b[Cl⁻]

3 ml of acetonitrile was added to a mixture of **1** (80 mg, 0.10 mmol) and AgCl (30.0 mg, 0.21 mmol, 2 eq.) in one portion at room temperature and the mixture was stirred for 16 h. Excess of silver chloride was separated by filtration and the solution was evaporated under reduced pressure to yield the crude product as a beige solid. The product was washed with 0.5 ml of toluene and dried in fine vacuum to obtain 72.0 mg (76%) of **3b** as a colorless solid.

^{1}H NMR (500.1 MHz, CD_3CN): δ [ppm] = 7.11 (s, 8H, Mes*H*-3,5), 7.08 (s, 4H, NC*H*), 3.04 (br, 2H C_2H_4), 2.86 (br, 2H, C_2H_4), 2.38 (s, 12H, Mes-C*H*$_3$), 2.08 (s, 12H, Mes-C*H*$_3$), 2.02 (s, 12H, Mes-C*H*$_3$).
^{13}C{^{1}H} NMR (125.8 MHz, CD_3CN): δ [ppm] = 146.79 (N*C*N), 142.35 (Mes*C*-1), 136.16 (Mes*C*-2,6), 131.77 (Mes*C*-4), 131.37 (Mes*C*-3,5), 131.04 (Mes*C*-3,5), 121.92 (N*C*H), 48.46 (C_2H_4), 21.31 (p-Mes*C*H$_3$), 18.96 (o-Mes*C*H$_3$).
^{29}Si NMR (99.4 MHz, CD_3CN): δ [ppm] = 20.18 (d, $^{1}J_{Si^{109}Ag}$ = 592.8 Hz), 20.18 (d, $^{1}J_{Si^{107}Ag}$ = 514.3 Hz).
MS (ESI^{+}) *m/z* calcd: 871.80; *m/z* found: 871.4
Elemental analysis: Sufficient elemental analysis could not be obtained due to high light sensitivity.
M.P.: 254.6 °C (decomposition)

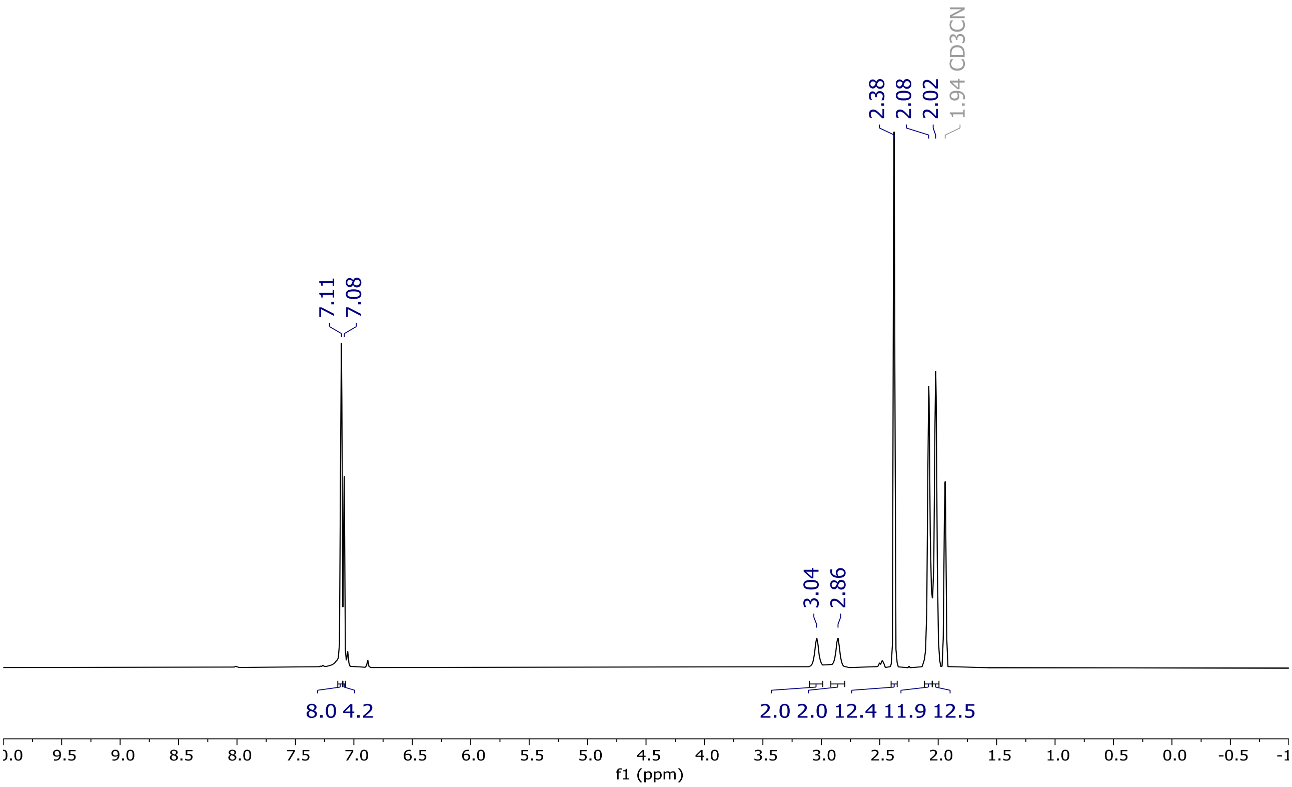

Figure S22 ^{1}H NMR spectrum of compound **3b** in CD_3CN at 300 K.

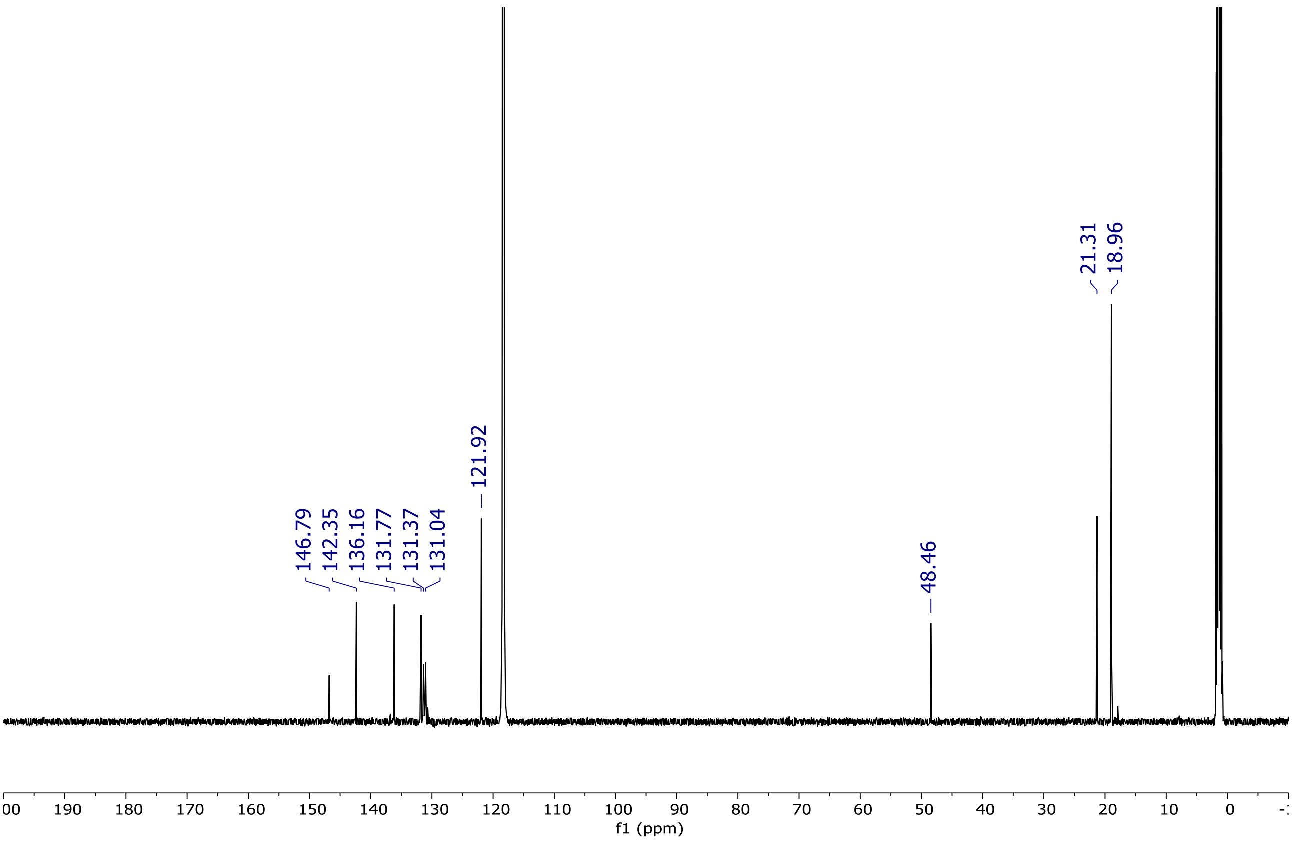

Figure S23 $^{13}C\{H\}$ NMR spectrum of compound **3b** in CD_3CN at 300 K.

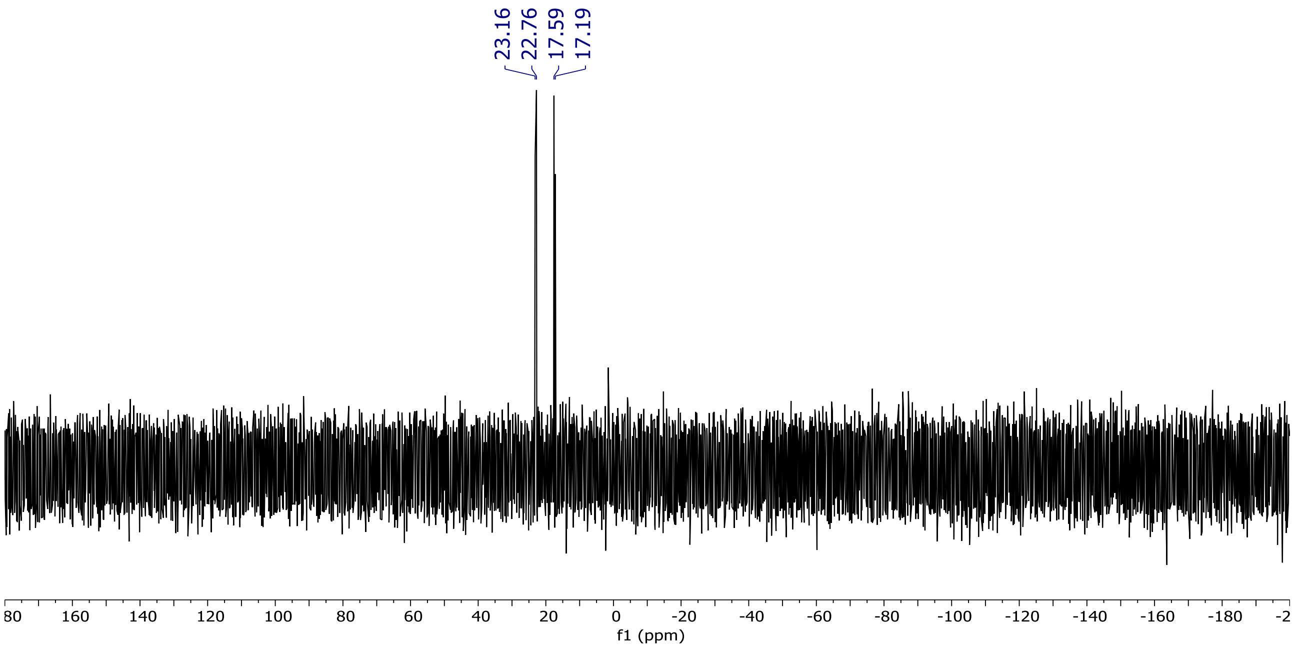

Figure S24 ^{29}Si NMR spectrum of compound **3b** in CD_3CN at 300 K.

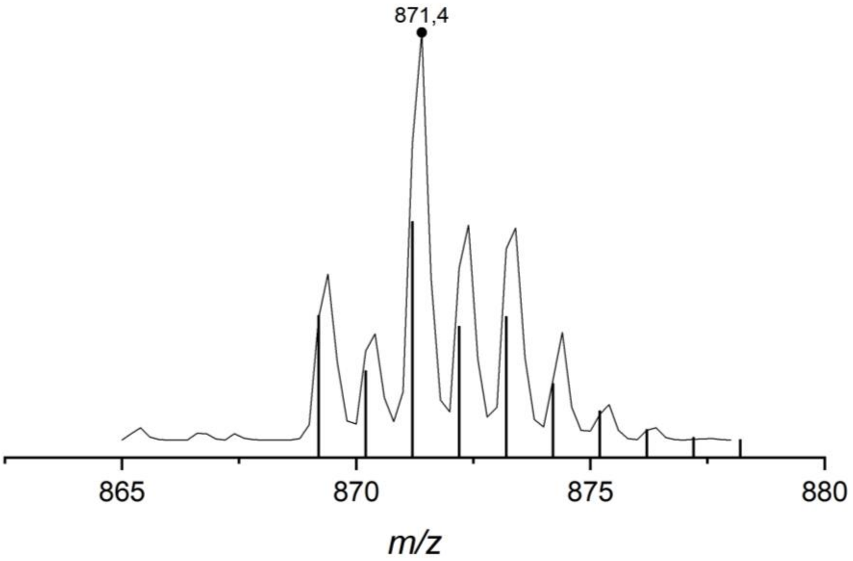

Figure S 25 ESI-MS spectrum (detail view) of **3b** (positive mode, 300 °C, –2500 V; line: measured spectrum; bars: simulated spectrum).

Procedure for the isolation of [Bis-NHI^{Mes}-SiCl(AuCl)]Cl (3c)

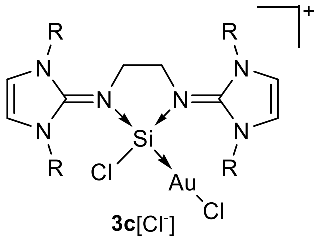

3c[Cl$^-$]

3 ml of acetonitrile was added to a mixture of **1** (80 mg, 0.10 mmol) and Me_2SAuCl (30.9 mg, 0.10 mmol, 1 eq.) in one portion at room temperature and the mixture was stirred for 1 h. Minor amount of insoluble residue was separated by filtration and the solution was evaporated under reduced pressure to yield the crude product as a slightly violet solid. The product was washed with 0.5 ml of toluene and dried in fine vacuum to obtain 92.0 mg (88%) of **3c** as a colorless solid.

^{1}H NMR (500.1 MHz, CD_3CN): *δ* [ppm] = 7.13 (s, 4H, NC*H*) , 7.09 (s, 4H, Mes*H*-3,5), 7.02 (s, 4H, Mes*H*-3,5), 3.14 (m, 2H C_2H_4), 2.86 (m, 2H, C_2H_4), 2.38 (s, 12H, Mes-C*H*$_3$), 2.16 (s, 12H, Mes-C*H*$_3$), 2.01 (s, 12H, Mes-C*H*$_3$).

^{13}C{^{1}H} NMR (125.8 MHz, CD_3CN): *δ* [ppm] = 146.29 (N*C*N), 142.13 (Mes*C*-1), 136.27 (Mes*C*-2,6), 136.16 (Mes*C*-2,6), 131.75 (Mes*C*-4), 131.14 (Mes*C*-3,5), 130.95 (Mes*C*-3,5), 122.32 (N*C*H), 48.44 (C_2H_4), 21.31 (p-Mes*C*H$_3$), 19.21 (o-Mes*C*H$_3$) , 18.95 (o-Mes*C*H$_3$).

^{29}Si NMR (99.4 MHz, CD_3CN): *δ* [ppm] = 18.20.

MS (ESI$^+$) *m/z* calcd: 959.31; *m/z* found: 959.2

Elemental analysis: [994.28] calcd: C 53.04, H 5.26, N 8.44; found: C 50.75, H 5.11, N 8.06. (Consistently low C values can be explained by the formation of silicon carbide.)

M.P.: 172.6 °C (decomposition)

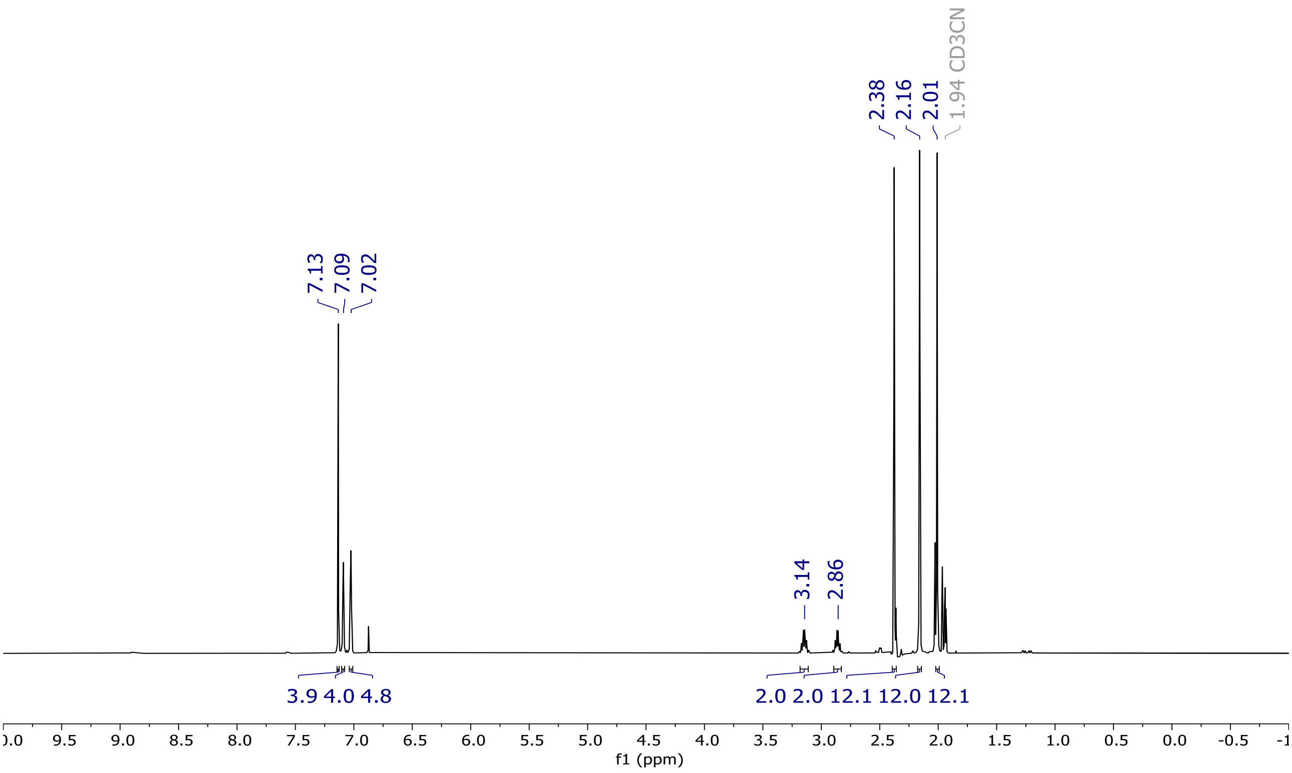

Figure S26 ^{1}H NMR spectrum of compound **3c** in CD_3CN at 300 K.

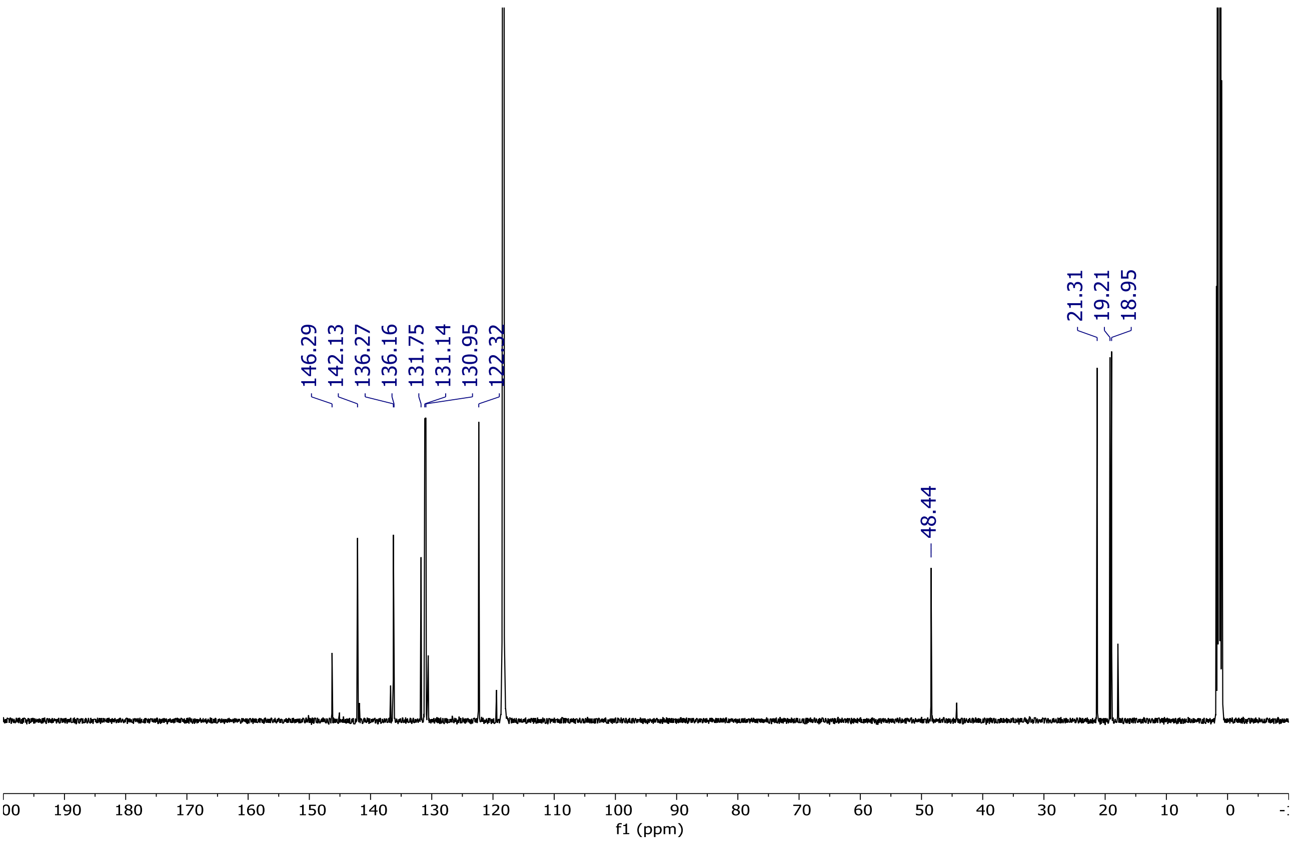

Figure S27 $^{13}C\{H\}$ NMR spectrum of compound **3c** in CD_3CN at 300 K.

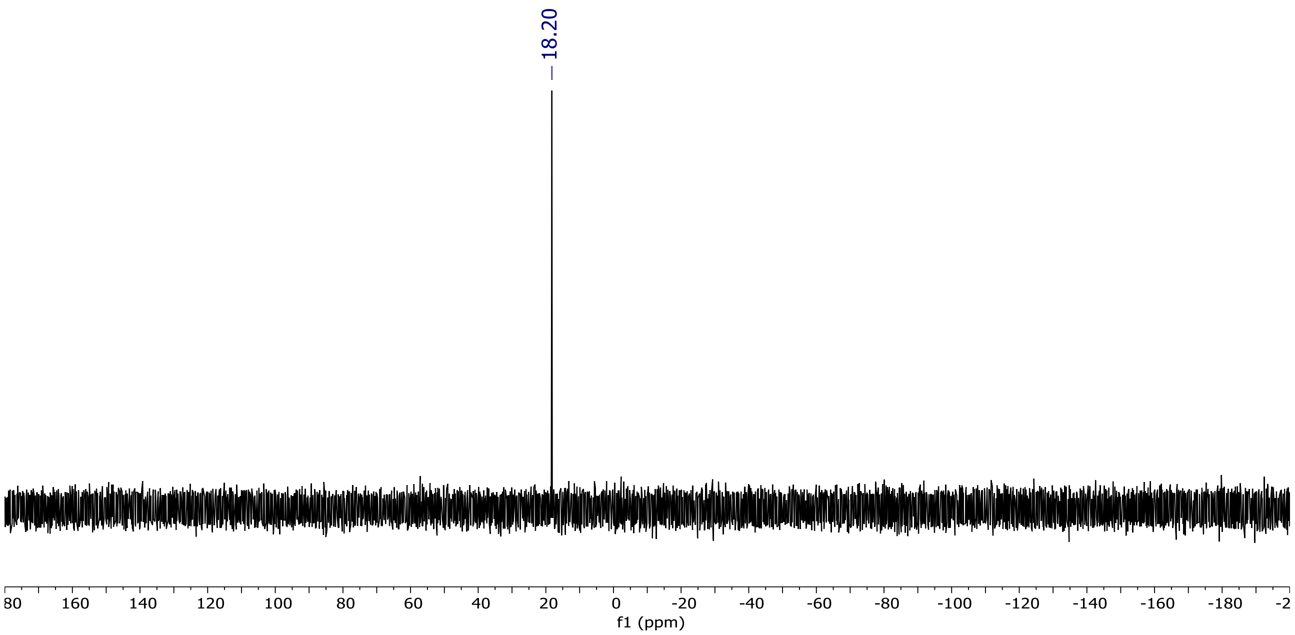

Figure S28 ^{29}Si NMR spectrum of compound **3c** in CD_3CN at 300 K.

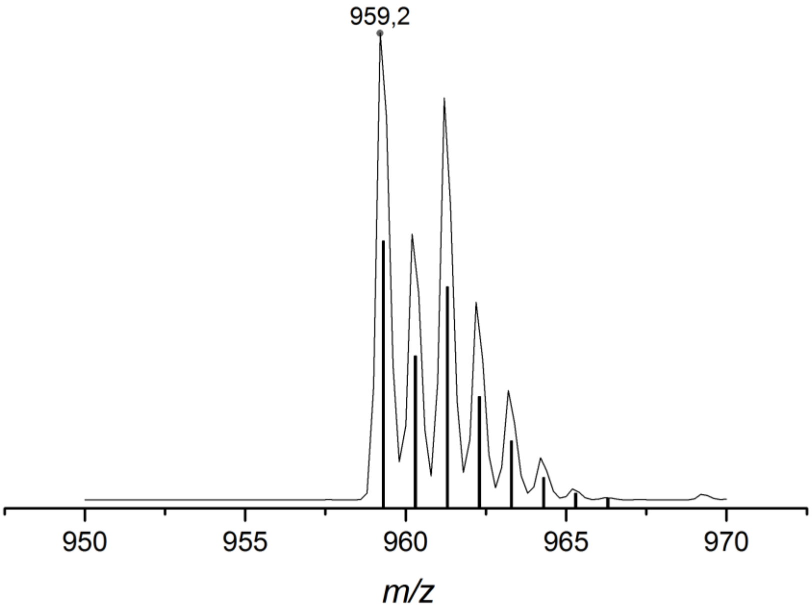

Figure S29. ESI-MS spectrum (detail view) of **3c** (positive mode, 300 °C, –2500 V; line: measured spectrum; bars: simulated spectrum).

Procedure for the isolation of Bis-NHI^{Mes}-SiClAuFe(CO)$_4$ (4)

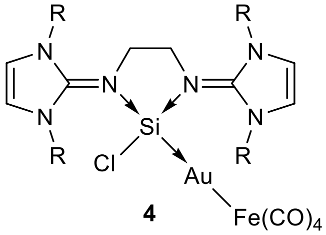

4

4 ml of acetonitrile was added to a mixture of **1** (100 mg, 0.13 mmol) and PPh_3AuCl (64.8 mg, 0.13 mmol, 1 eq.) in one portion at room temperature and the mixture was stirred for 30 min under the exclusion of light. The stirring solution is cooled to -30 °C and $K_2Fe(CO)_4$ (32.2 mg, 0.13 mmol, 1 eq.) suspended in 2 ml acetonitrile is added expeditiously dropwise. The mixture was allowed to warm to room temperature and stirred for additional 10 min. Precipitated KCl was separated from the yellow solution by filtration. The filtrate was concentrated under reduced pressure to ca. 2 ml. The product solution was stored at +5 °C over 2 days to yield **4** as yellow crystals. The Product was separated from the mother liquor and dried in fine vacuum to obtain 80 mg (56%) of **4** as a yellow crystalline solid. X-ray quality crystals of **4** were obtained from a concentrated acetonitrile solution of **4** after 2 days.

^{1}H NMR (500.1 MHz, pyr-d5): δ [ppm] = 7.36 (s, 4H, NC*H*) , 6.78 (s, 4H, Mes*H*-3,5), 6.70 (s, 4H, Mes*H*-3,5), 3.38 (m, 2H C_2H_4), 2.94 (m, 2H, C_2H_4), 2.58 (s, 12H, Mes-CH_3), 2.36 (s, 12H, Mes-CH_3), 2.19 (s, 12H, Mes-CH_3).

^{13}C{^{1}H} NMR (125.8 MHz, pyr-d5): δ [ppm] = 228.95 (*C*O),147.52 (N*C*N), 140.75 (Mes*C*-1), 136.20 (Mes*C*-2,6), 132.36 (Mes*C*-4), 130.96 (Mes*C*-3,5), 130.25 (Mes*C*-3,5), 121.70 (N*C*H), 48.49 (C_2H_4), 21.54 (p-Mes*C*H_3), 19.36 (o-Mes*C*H_3), 19.26 (o-Mes*C*H_3).

^{29}Si NMR (99.4 MHz, pyr-d5): δ [ppm] = 67.56.

IR (ATR, neat) [cm^{-1}]: ʋ(CO) = 1924, 1835, 1811, 1796

Elemental analysis: [1092.25] calcd: C 52.73, H 4.79, N 7.69; found: C 51.37, H 5.06, N 7.71. (Consistently low C values can be explained by the formation of silicon carbide.)

M.P.: 155.8 °C (decomposition, color change to dark brown)

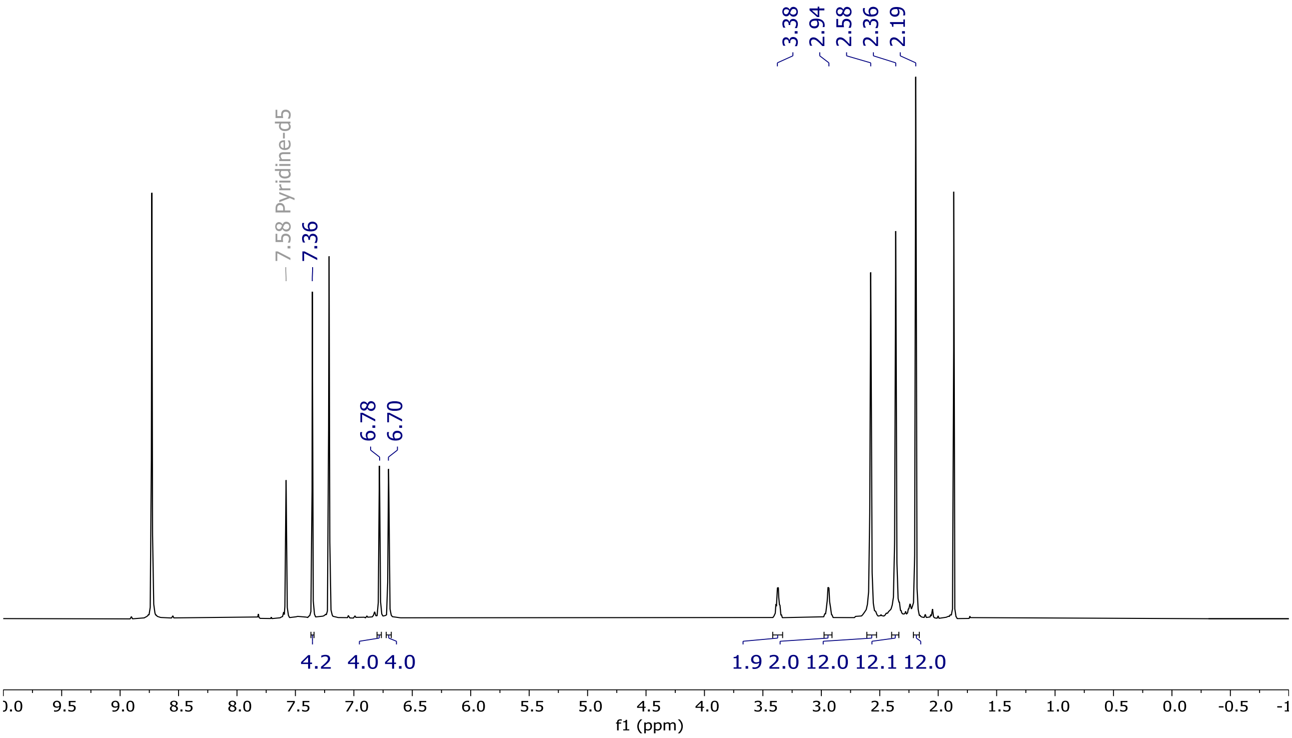

Figure S30 ^{1}H NMR spectrum of compound **4** in pyr-d5 at 300 K. Acetonitrile from synthesis is contained.

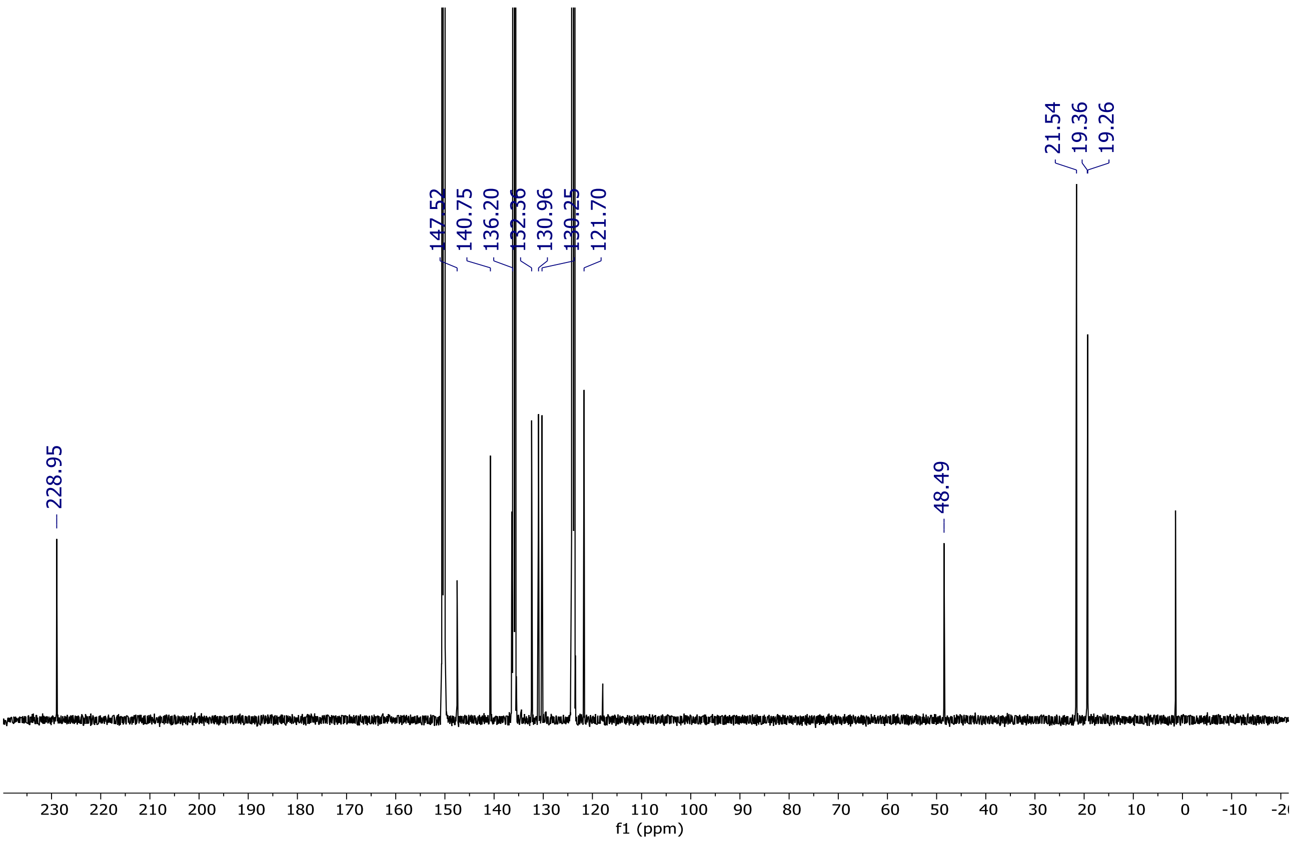

Figure S31 ^{13}C{H} NMR spectrum of compound **4** in pyr-d5 at 300 K. Acetonitrile from synthesis is contained.

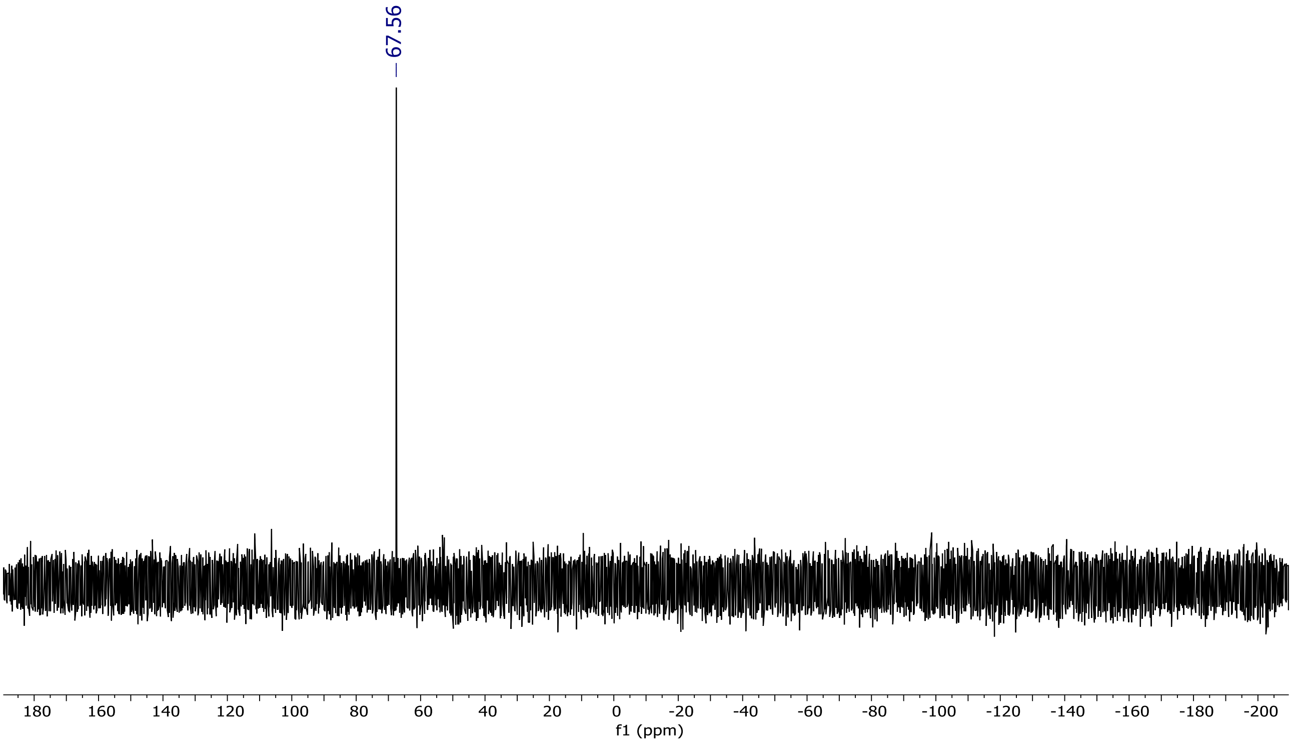

Figure S32 ^{29}Si NMR spectrum of compound **4** in pyr-d5 at 300 K.

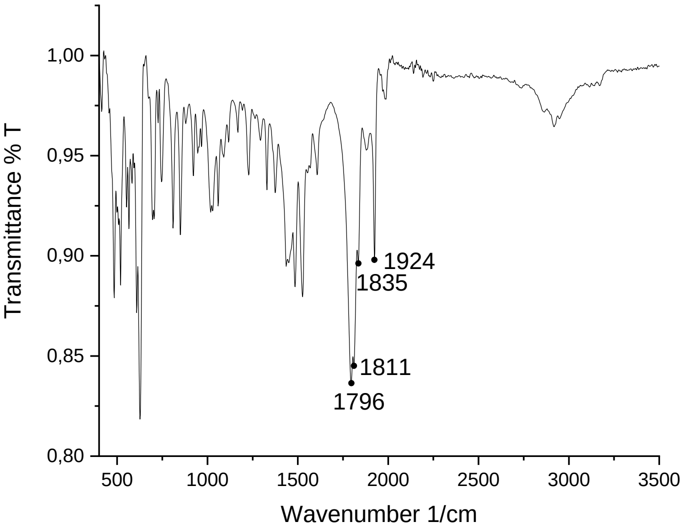

Figure S33 IR spectrum of compound **4**. (ATR, neat).

2. Crystallographic Details

General considerations: Single crystal diffraction data were recorded on a Bruker Photon CMOS system equipped with a Helios optic monochromator and a Mo IMS microsource (λ = 0.71073 Å). The data collection was performed, using the APEX III software package[S5] on single crystals coated with Fomblin ® Y as perfluorinated ether. The single crystal was picked on a micro sampler, transferred to the diffractometer and measured frozen under a stream of cold nitrogen. A matrix scan was used to determine the initial lattice parameters. Reflections were merged and corrected for Lorenz and polarization effects, scan speed, and background using SAINT.[S6] Absorption corrections, including odd and even ordered spherical harmonics were performed using SADABS.[S6] Space group assignments were based upon systematic absences, E statistics, and successful refinement of the structures. Structures were solved by direct methods with the aid of successive difference Fourier maps, and were refined against all data using the APEX III software in conjunction with SHELXL-2014[S7] and SHELXLE.[S8] H atoms were placed in calculated positions and refined using a riding model, with methylene and aromatic C–H distances of 0.99 and 0.95 Å, respectively, and Uiso(H) = 1.2·Ueq(C). Non-hydrogen atoms were refined with anisotropic displacement parameters. Full-matrix least-squares refinements were carried out by minimizing $\Sigma w(F_o^2-F_c^2)^2$ with the SHELXL-97 weighting scheme.[S9] Neutral atom scattering factors for all atoms and anomalous dispersion corrections for the non-hydrogen atoms were taken from International Tables for Crystallography.[S10] The images of the crystal structures were generated by Mercury.[S11] The CCDC numbers CCDC-(2085156 - 2085158) contain the supplementary crystallographic data for the structures **1**, **2a** and **4**. These data can be obtained free of charge from the Cambridge Crystallographic Data Centre via https://www.ccdc.cam.ac.uk/structures/.

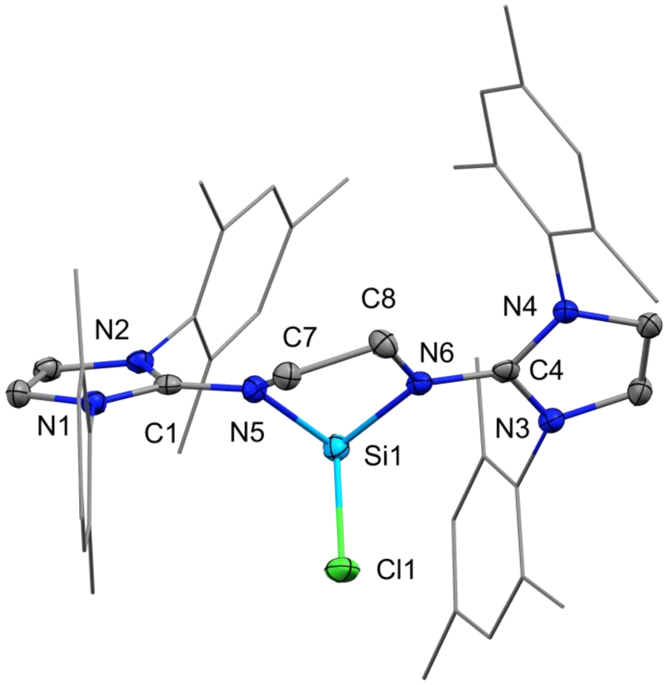

Figure S34 Solid state plot of the molecular structure of **1**. Thermal ellipsoids are set to 50% probability level. Hydrogens, the counter anion and lattice solvent are omitted for clarity; mesityl-substituents are partly drawn as wireframe for simplicity. Selected bond lengths [Å] and angles [°]: Si1–Cl1 2.2374(8), Si1–N5 1.8290(18), Si1–N6 1.8617(18), C1–N5 1.337(3), C4–N6 1.336(3), Cl1–Si1–N5 95.07(6), N5–Si1–N6 84.00(8), Cl1–Si1–N6 94.91(6).

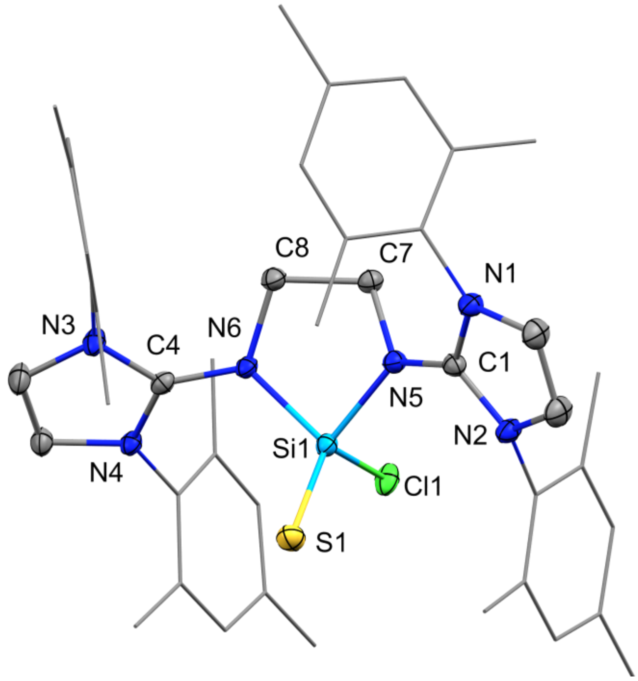

Figure S35 Solid state plot of the molecular structure of **2a**. Thermal ellipsoids are set to 50% probability level. Hydrogens, solvent molecules, and the counter anion are omitted for clarity; mesityl-substituents are partly drawn as wireframe for simplicity. Selected bond lengths [Å] and angles [°]: Si1–S1 1.9740(6), Si1–Cl1 2.0678(6), Si1–N5 1.7839(12), Si1–N6 1.7972(14), C1–N5 1.3446(19), N6–C4 1.356(2), N5–Si1–N6 89.89(6), N5–Si1–S1 120.22(5), S1–Si1–Cl1 117.05(3), N5–Si1–Cl1 101.96(5).

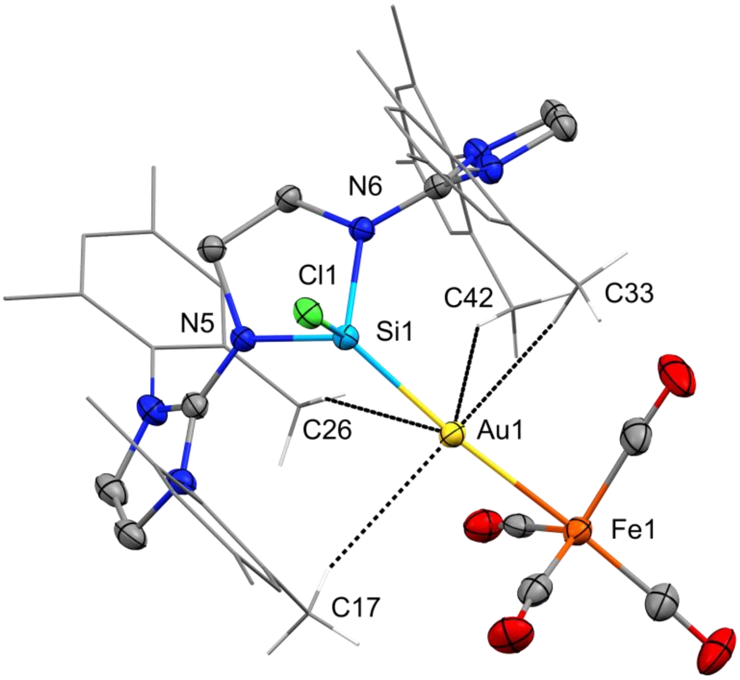

Figure S36 Solid state plot of the molecular structure of **4**. Thermal ellipsoids are set to 50% probability level; Hydrogens are omitted for clarity; mesityl-substituents are partly drawn as wireframe for simplicity. Selected bond lengths [Å] and angles [°]: Au1–Si1 2.2676(9), Au1–Fe1 2.5305(6), Si1–N5 1.793(3), Si1–N6 1.819(2), Si1–Cl1 2.1076(11), C1–N5 1.352(5), C4–N6 1.355(4), Si1–Au1–Fe1 173.65(3), Cl1–Si1–Au1 119.02(5), N5–Si1–N6 87.80(13).

Table S1 Crystal data and structure refinement for compound **1**, **2a**, **4**.

Compound #	1	2a	4
Chemical formula	$C_{44} H_{52} Cl_2 N_6 Si, 2(C_6 H_4 F_2)$	$C_{44} H_{52} Cl_2 N_6 S Si, 3 (C_2 H_3 N)$	$C_{48} H_{52} Au Cl Fe N_6 O_4 Si$
Formula weight	992.09 g/mol	919.13 g/mol	1093.32 g/mol
Temperature	100 K	100 K	100 K
Wavelength	0.71073 Å	0.71073 Å	0.71073 Å
Crystal size	0.378 x 0.296 x 0.130 mm	0.273 x 0.211 x 0.182 mm	0.176 x 0.165 x 0.091 mm
Crystal habit	clear colorless fragment	clear colorless fragment	clear light yellow fragment
Crystal system	triclinic	triclinic	monoclinic
Space group	P -1	P -1	C 2/c
Unit cell dimensions	a = 14.0779(12) Å; α = 76.724(4)°	a = 12.2139(11) Å; α = 70.258(3)°	a = 30.060(2) Å; α = 90°
	b = 14.3401(12) Å; β = 71.447(4)°	b = 14.4858(13) Å; β = 84.626(3)°	b = 16.7584(11) Å; β = 121.366(2)°
	c = 14.7770(12) Å; γ = 67.418(4)°	c = 15.2925(13) Å; γ = 80.130(3)°	c = 23.3357(16) Å; γ = 90°
Volume	2591.5(4) $Å^3$	2507.1(4) $Å^3$	10037.6(12) $Å^3$
Z	2	2	8
Density (calculated)	1.271 g/cm^3	1.218 g/cm^3	1.447 g/cm^3
Radiation source	TXS rotating anode	TXS rotating anode	IMS microsource
Theta range for data collection	2.26 to 25.39 °	2.39 to 25.42 °	2.00 to 25.35°
Index ranges	-16<=h<=16, -17<=k<=17, -17<=l<=17	-14<=h<=14, -17<=k<=17, -18<=l<=18	-36<=h<=36, -20<=k<=20, -28<=l<=28
Reflections collected	45078	131380	149934
Independent reflections	9486	9241	9204
Completeness	0.996	0.998	0.999
Absorption correction	Multi-Scan	Multi-Scan	Multi-Scan
Max. and min. transmission	0.5463 and 0.7452	0.7144 and 0.7452	0.6606 and 0.7452
Refinement method	Full-matrix least-squares on F^2	Full-matrix least-squares on F^2	Full-matrix least-squares on F^2
Function minimized	Δ $w(F_o^2 - F_c^2)^2$	Δ $w(F_o^2 - F_c^2)^2$	Δ $w(F_o^2 - F_c^2)^2$
Data / restraints / parameters	9486 / 0 / 634	9241 / 0 / 583	9204 / 0 / 571
Goodness-of-fit on F^2	1.028	1.038	1.139
Final R indices [I>2sigma(I)]	R1 = 0.0457, wR2 = 0.0981	R1 = 0.0346, wR2 = 0.0930	R1 = 0.0213, wR2 = 0.0467
R indices (all data)	R1 = 0.0747, wR2 = 0.1092	R1 = 0.0386, wR2 = 0.0961	R1 = 0.0312, wR2 = 0.0533
Largest diff. peak and hole	0.324 and -0.352 $eÅ^{-3}$	0.313 and -0.351 $eÅ^{-3}$	0.957 and -0.682 $eÅ^{-3}$

3. Computational Details

All calculations were performed with ORCA v.4.0.1.[S12, 13] Two computational strategies were applied, which both gave consistent results:

1) The geometric parameters were optimized using the PBE0 functional,[S14, 15] with dispersion correction D3(BJ)[S16, 17] and the def2-SVP basis set.[S18] For Si, Au, and Fe, the def2-TZVP basis set and the def2-ECP (for Au) were used.[S19, 20] Tighter than default scf ("*tightscf*") and optimization criteria ("*tightopt*") were chosen in conjunction with finer than default grid values ("*grid7*"; "*nofinalgrid*") and WITHOUT the RIJCOSX approximation. The optimized geometric parameters were verified as true minima by the absence of negative eigenvalues in the harmonic vibrational frequency analysis. For the analysis of the electronic structure, single-point calculations were performed (PBE0-D3BJ/def2-TZVPP//PBE0-D3BJ/def2-SVP). The population analysis was performed using Knizia's Intrinsic Bond Orbitals.[S21] Other localization methods (Foster-Boys, Pipek-Mezey) led to similar results. The calculations for the Mößbauer parameters followed a procedure developed by F. Neese and coworkers.[S22, 23] The calculation of the ^{57}Fe Mößbauer parameters was performed with the TPSSh functional and without the RIJCOSX approximation, the core properties basis set CP(PPP)[S24] for iron and the DKH-def2-TZVPP basis set for all other atoms, the second order Douglas-Kroll-Hess method,[S25] and even tighter scf ("*verytightscf*") and grid setting for iron, gold and silicon (*"SpecialGridIntAcc 7"*) and the other atoms ("*grid*6", "*nofinalgrid*"). The ^{57}Fe Mößbauer isomer shifts were calibrated using the extended ("whole") calibration parameters according to F. Neese.[S23] This calibration set includes previously reported[S22, 26] calibration sets. Modeling of the Mößbauer spectrum with the BP86 and B3LYP functionals led to comparable spectroscopic parameters. Accordingly, similar spectroscopic parameters were obtained for the solid-state structure with optimized positions of hydrogen atoms as coordinates.

2) Scalar relativistic calculations with the Zeroth Order Regular Approximation (ZORA)[S27, 28] and the BP86 functional were performed (ZORA-BP86-D3BJ/def2-TZVPP//ZORA-BP86-D3BJ/def2-SVP). For Au, and Fe, the SARC all-electron basis set were used, for Si the ZORA-def2-TZVP basis set. A comparable fit as obtained with strategy 1 was obtained for the structural parameters in the solid state (Table S2), whereas the vibrational spectra (frequencies were scaled by 0.981 according to ref[S29]) were in better agreement with the experiment and are thus shown in the manuscript. The IBOs (Figure S37), which could not be obtained at the ZORA level, were calculated at the BP86/def2-TZVPP(def2-ECP)//ZORA-BP86-D3BJ/def2-SCP level of theory and are essentially equivalent to the ones obtained with PBE0.

Table S2 Benchmark of structural parameters of **4**. Bond lengths are given in [Å], angles in [°].

	**Solid State**	**PBE0**	**BP86**
Si-Cl	2.1076	2.13	2.145
Si-Au	2.2676	2.27	2.261
Au-Fe	2.5305	2.5	2.504
CO-HCH$_2$Mes	2.579	2.896	2.51
Si-Au-Fe	173.65	168.6	169.2

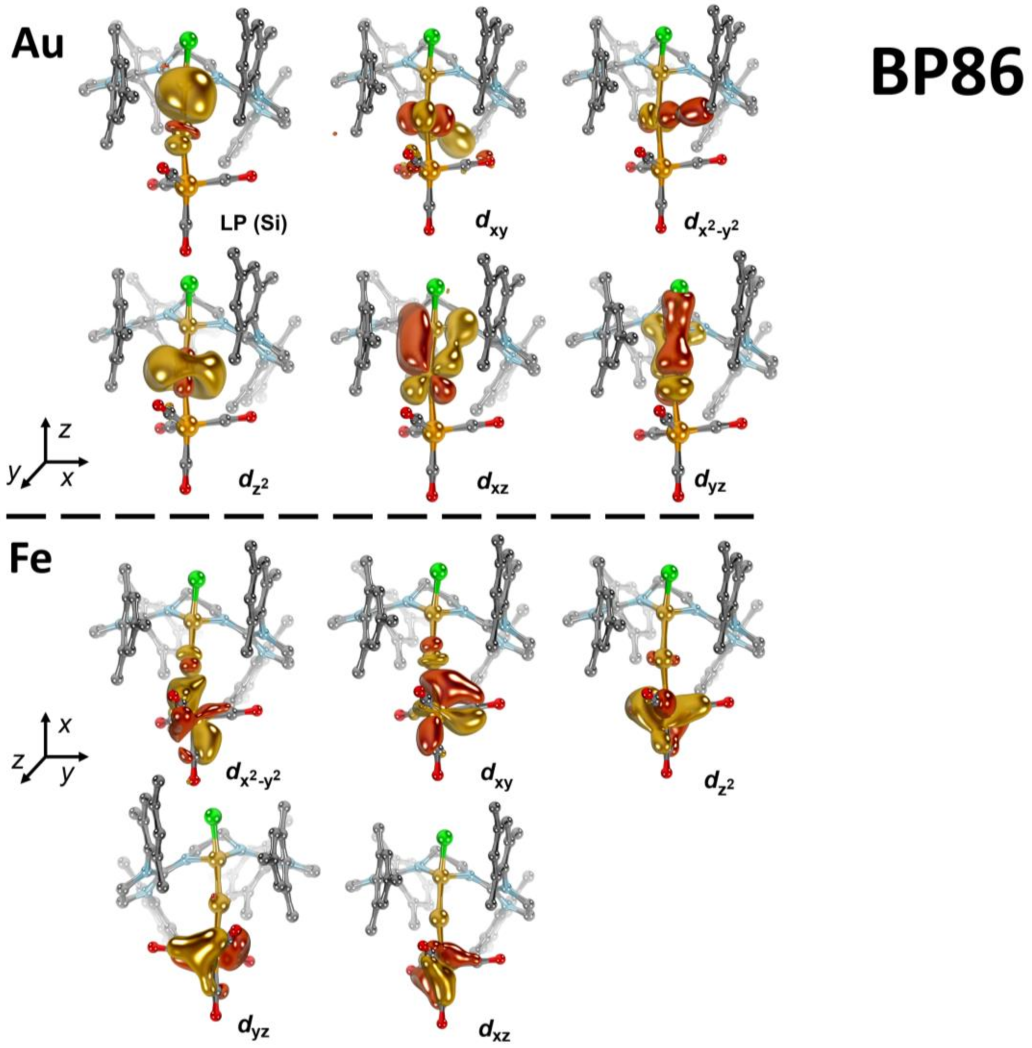

Figure S37 IBOs as obtained with BP86.

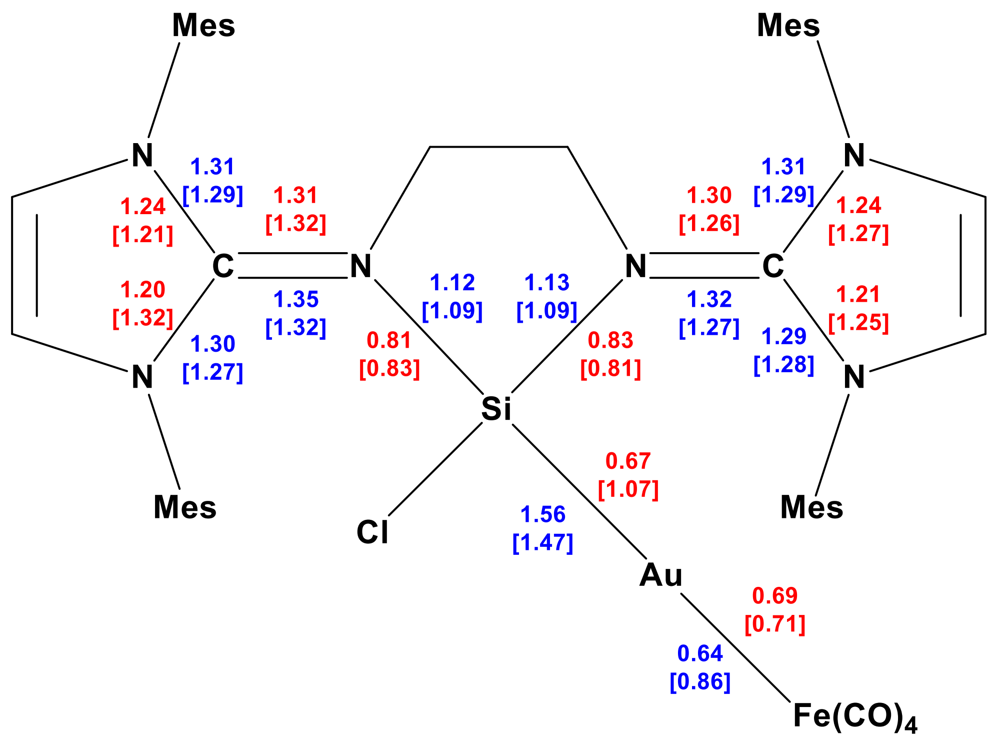

Figure S38 Bond orders in **4**. Mayer bond orders in red (PBE0 [ZORA-PB86]) and Löwdin bond orders in blue (PBE0 [ZORA-PB86]).

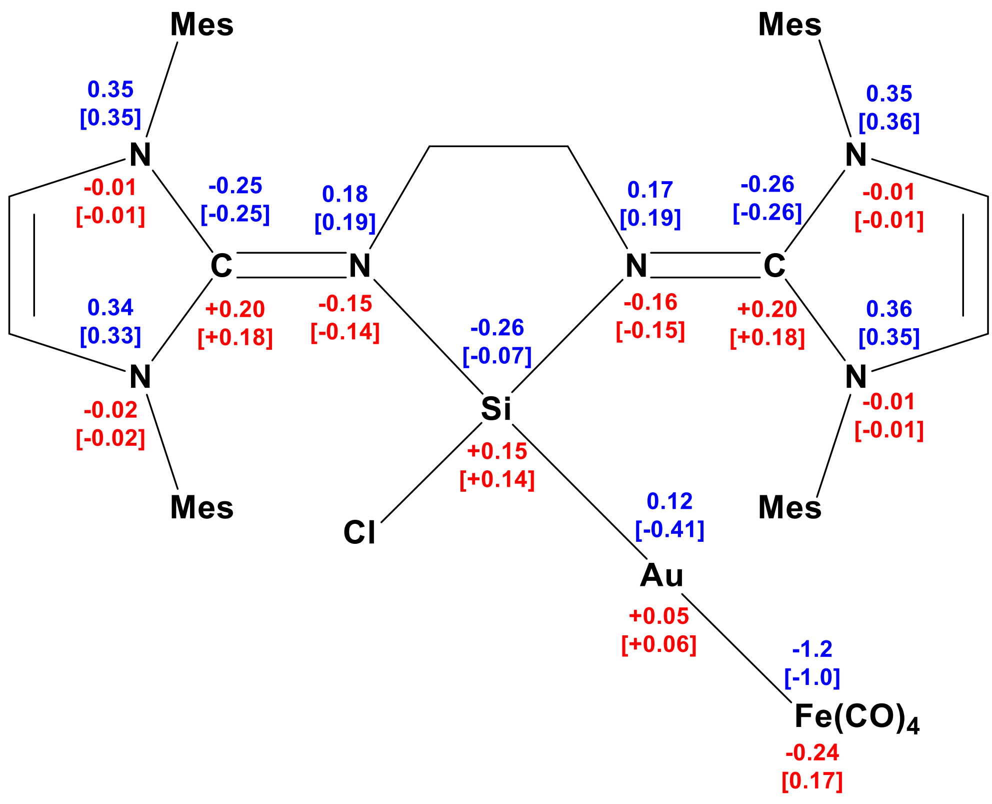

Figure S39 Calculated partial charges in **4**. Hirshfeld partial charges in red (PBE0 [ZORA-PB86]) and Löwdin partial charges in blue (PBE0 [ZORA-PB86]).

Table S3 Energies of calculated compounds.

BP				
**Compound**	***E* [Eh]**	***H* [Eh]**	***G* [Eh]**	**NImag**
**$Fe(CO)_4^{2-}$**	-1731.583664	-1731.541484	-1731.585866	0
**A**	-22334.061991	-22333.602426	-223337.039014	0
**$NHCMesFe(CO)_4$**	-2655.992479	-2655.533689	-2655.628490	0
**JFe**	-3138.766511	-3138.152546	-3138.263869	0
**4**	-24199.554221	-24198.599820	-24198.757207	0
**PBE0**				
**$Fe(CO)_4^{2-}$**	-1715.978536	-1715.935089	-1715.979077	0
**$NHCMesFe(CO)_4$**	-2638.600065	-2638.128933	-2638.222504	0
**4**	-4634.120518	-4633.139796	-4,633.294931	0

XYZ coordinates

NHC^Mes_Fe(CO)$_4$_BP86

C	-1.13671	0.89300	-2.88598
C	-1.20232	-0.16370	-2.02235
N	-1.20844	0.37334	-0.73444
C	-1.15006	1.75225	-0.75487
N	-1.10469	2.04727	-2.10260
H	-1.11105	0.94207	-3.97396
H	-1.24641	-1.23899	-2.19132
Fe	-1.35595	3.00339	0.75967
C	-3.09381	2.85119	0.43966
O	-4.24747	2.76999	0.25085
C	-0.51660	1.96259	1.95013
O	0.01807	1.42154	2.83674
C	-1.72395	4.11458	2.09189
O	-1.97714	4.84293	2.96362
C	-0.37188	4.31064	0.03240
O	0.25266	5.24492	-0.28647
C	-0.86210	3.34162	-2.68064
C	0.48632	3.70119	-2.91582
C	-1.93817	4.18168	-3.02903
C	0.74076	4.95311	-3.49385
C	-1.62704	5.43183	-3.59925
C	-0.30202	5.83927	-3.83167
H	1.78247	5.25517	-3.66505
H	-2.45147	6.10572	-3.86895
C	-1.09981	-0.46404	0.42972
C	-2.25546	-0.88823	1.11506
C	0.20062	-0.86655	0.81648
C	-2.07488	-1.71387	2.24216
C	0.32415	-1.69621	1.94021
C	-0.80104	-2.12087	2.67522
H	-2.96260	-2.04748	2.79643
H	1.32769	-2.00170	2.26468
C	-3.37314	3.78844	-2.79005
H	-4.01342	4.11381	-3.62831
H	-3.49298	2.70127	-2.66057
H	-3.75995	4.26345	-1.87124
C	0.00466	7.20613	-4.39657
H	0.85326	7.17310	-5.10250
H	-0.86581	7.63485	-4.92183
H	0.28145	7.90656	-3.58539
C	1.60915	2.79245	-2.48216
H	1.61217	2.68427	-1.38113
H	1.50918	1.77551	-2.90327
H	2.58682	3.19896	-2.78725
C	-3.63929	-0.46980	0.68981
H	-3.66862	-0.12270	-0.35509
H	-4.35294	-1.30449	0.80147
H	-4.00313	0.36563	1.31361
C	1.41325	-0.34917	0.08403
H	2.33647	-0.80509	0.47645
H	1.36207	-0.54803	-1.00189
H	1.49527	0.74750	0.20484
C	-0.63364	-2.96304	3.91786
H	-1.56048	-3.50582	4.17054
H	0.17940	-3.70129	3.80208
H	-0.37526	-2.32544	4.78503

A_BP86

C	-0.96231	0.00376	-3.86231
C	-1.03563	-1.05274	-2.98751
N	-0.77523	-0.53378	-1.71674
C	-0.54048	0.82313	-1.75989
N	-0.65792	1.12916	-3.10047
H	-1.09857	0.05347	-4.94253
H	-1.24845	-2.10928	-3.15005
Fe	-1.42533	4.03591	1.16713
C	-2.97753	3.37061	0.67985
O	-4.02981	2.92882	0.34072

C	-0.43906	3.01104	2.22561
O	0.19668	2.36395	2.98766
C	-1.90246	5.28114	2.31022
O	-2.22377	6.11543	3.08061
C	-0.64474	5.03400	-0.06609
O	-0.14531	5.75493	-0.86416
C	-0.60839	2.50198	-3.52524
C	0.65405	3.09066	-3.74724
C	-1.80406	3.25304	-3.51912
C	0.70110	4.47412	-3.98126
C	-1.70250	4.63404	-3.76047
C	-0.46314	5.26499	-3.96679
H	1.67863	4.95675	-4.11630
H	-2.61482	5.24341	-3.71331
C	-0.83278	-1.25160	-0.47606
C	-2.04859	-1.27249	0.24222
C	0.34810	-1.84492	0.01610
C	-2.05931	-1.94879	1.47618
C	0.28538	-2.50802	1.25398
C	-0.90813	-2.56710	1.99912
H	-2.99148	-1.96583	2.05713
H	1.19862	-2.96632	1.65818
C	-3.12276	2.62923	-3.13857
H	-3.96717	3.22726	-3.52108
H	-3.22492	1.59529	-3.51145
H	-3.21479	2.59540	-2.03486
C	-0.37131	6.76874	-4.04045
H	0.50792	7.10219	-4.62006
H	-1.27743	7.21726	-4.48486
H	-0.26964	7.15423	-3.00790
C	1.91047	2.26662	-3.61452
H	2.05183	1.97699	-2.55498
H	1.86005	1.33078	-4.20056
H	2.79668	2.83784	-3.93854
C	-3.26284	-0.53405	-0.25728
H	-3.39071	-0.63524	-1.34916
H	-4.17898	-0.89130	0.24188
H	-3.16531	0.54982	-0.04586
C	1.64254	-1.69599	-0.74424
H	2.46101	-2.24496	-0.24920
H	1.55619	-2.05857	-1.78510
H	1.92211	-0.62677	-0.80143
C	-0.93861	-3.22461	3.36037
H	-1.91505	-3.70093	3.56023
H	-0.15201	-3.99350	3.45916
H	-0.77076	-2.47437	4.15706
Au	-0.64273	2.19625	-0.31186

$Fe(CO)_4^{2-}$_BP86

O	2.40120	4.17319	-1.41987
Fe	3.51635	3.73951	1.25200
O	1.80767	1.99028	2.87049
O	6.20225	2.66357	0.82925
C	2.83629	3.99746	-0.32728
C	2.50041	2.69275	2.20649
C	5.10384	3.08082	1.01019
C	3.70589	5.24208	2.09971
O	3.85431	6.26844	2.68067

4_BP86

Cl	3.17654	9.33918	2.11010
Si	3.95346	7.38073	2.51336
Au	3.63797	5.35049	1.56913
O	2.46083	4.22220	-1.56479
Fe	3.27458	2.94628	0.96937
N	7.07532	7.53591	1.12279
C	6.83230	7.65862	2.47161
N	8.05047	7.48035	3.09619
O	6.03283	2.79939	2.00579
C	8.43222	7.27672	0.91328
H	8.82366	7.14426	-0.09354
N	1.24379	6.48730	4.23207

O	1.19441	3.01412	3.05006
C	9.04312	7.25097	2.13204
H	5.84374	2.50086	6.66652
C	8.84594	8.15934	5.32099
C	4.31673	3.92309	6.12274
C	7.09395	4.96148	4.05750
H	6.94254	3.99593	4.56022
H	7.71703	4.77611	3.16540
H	6.10633	5.28463	3.67571
C	4.03877	3.32446	4.77821
H	4.78510	2.56652	4.49798
H	4.01966	4.07815	3.96681
H	3.03968	2.85381	4.74410
C	8.45702	6.41551	8.72425
H	8.93071	7.24534	9.27481
H	9.02585	5.49326	8.94237
H	7.43859	6.26889	9.12812
C	6.48962	5.10784	-0.36824
H	6.71942	4.94503	0.69748
H	7.44367	5.07450	-0.93041
H	5.86843	4.25562	-0.68677
C	5.49302	8.84356	-0.21513
C	9.37006	9.45366	4.74709
H	9.65766	10.15338	5.54853
H	8.61652	9.95004	4.10801
H	10.25968	9.28855	4.11115
C	2.81464	3.73722	-0.54333
C	4.93155	2.88635	1.56927
C	2.03206	3.00009	2.20910
C	3.12220	1.27086	0.44605

NHC^{Mes}_Fe(CO)$_4$_PBE0

C	-1.07527	0.93520	-2.81878
C	-1.16910	-0.10641	-1.96187
N	-1.15091	0.42668	-0.68488
C	-1.05484	1.79251	-0.70973
N	-1.00556	2.08029	-2.04324
H	-1.05113	0.97859	-3.90357
H	-1.24764	-1.17574	-2.13431
Fe	-1.20591	3.03606	0.83297
C	-2.93378	2.99721	0.45270
O	-4.05595	2.99243	0.17798
C	-0.17286	2.00759	1.86983
O	0.53107	1.45085	2.59063
C	-1.58179	3.96066	2.29602
O	-1.86870	4.53994	3.24350
C	-0.33022	4.42668	0.13498
O	0.20984	5.39390	-0.17310
C	-0.82361	3.36411	-2.64995
C	0.48808	3.82878	-2.83216
C	-1.93605	4.07437	-3.12393
C	0.66499	5.06271	-3.45874
C	-1.70346	5.31111	-3.73105
C	-0.41785	5.82971	-3.89463
H	1.68139	5.44113	-3.60087
H	-2.55963	5.88667	-4.09534
C	-1.09502	-0.42725	0.46683
C	-2.26007	-0.77190	1.16394
C	0.15913	-0.96149	0.81454
C	-2.13424	-1.64823	2.24859
C	0.22834	-1.82494	1.90410
C	-0.90616	-2.17573	2.64157
H	-3.03600	-1.92897	2.80095
H	1.20127	-2.23318	2.19238
C	-3.33806	3.56246	-2.97883
H	-3.96225	3.89816	-3.81822
H	-3.38256	2.46621	-2.93053
H	-3.79941	3.93978	-2.05471
C	-0.21853	7.19404	-4.48936
H	0.79938	7.32563	-4.88403
H	-0.92996	7.39153	-5.30415
H	-0.38254	7.96987	-3.72310

C	1.66426	3.05523	-2.31669
H	1.69114	3.08374	-1.21479
H	1.62345	1.99500	-2.61055
H	2.60404	3.48093	-2.69237
C	-3.61029	-0.23053	0.80941
H	-3.66452	0.12484	-0.22788
H	-4.38587	-0.99496	0.96208
H	-3.85964	0.62761	1.45105
C	1.40077	-0.58494	0.06421
H	2.27739	-1.08528	0.49529
H	1.33930	-0.86475	-0.99963
H	1.57306	0.50217	0.10638
C	-0.78374	-3.08036	3.83219
H	-1.76214	-3.45108	4.17047
H	-0.14714	-3.95014	3.61050
H	-0.31832	-2.54379	4.67506

$Fe(CO)_4^{2-}$_PBE0

O	2.32057	4.21373	-1.44848
Fe	3.53460	3.60616	1.16569
O	1.80813	1.94038	2.86412
O	6.25538	2.56253	0.85713
C	2.78911	3.95780	-0.43811
C	2.48548	2.58707	2.20233
C	5.18225	2.95012	0.98421
C	3.71171	5.37113	2.14870
O	3.84097	6.65917	2.86608

4_PBE0

Cl	3.16913	9.24315	2.08389
Si	3.93537	7.30548	2.52566
Au	3.63773	5.27963	1.54579
O	2.75358	4.10655	-1.65314
Fe	3.34547	2.87419	0.93223
N	7.05822	7.54379	1.18235
C	6.79617	7.62627	2.51344
N	7.99368	7.46158	3.14738
O	6.05499	2.78073	2.01789
C	8.41198	7.32088	0.98764
H	8.81958	7.21951	-0.01272
N	1.20211	6.46717	4.23538
O	1.25397	2.97927	2.96235
C	8.99789	7.27960	2.20234
H	10.02740	7.11497	2.50363
N	2.43893	5.50770	5.75083
O	3.10411	0.09190	0.12571
C	2.46187	6.42736	4.74727
N	5.60644	7.83505	3.08398
C	0.40567	5.54326	4.89299
H	-0.63157	5.39502	4.61273
N	3.49247	7.17286	4.31499
C	1.16569	4.95683	5.83941
H	0.94903	4.16738	6.55172
C	5.45893	8.48698	4.38433
H	4.96730	9.46074	4.21960
H	6.41292	8.65677	4.89167
C	0.64762	7.49963	3.40612
C	0.52909	8.78305	3.96429
C	1.03381	9.09358	5.34057
H	0.58376	10.02032	5.72069
H	2.12675	9.23469	5.32108
H	0.81137	8.28799	6.05707
C	-0.03886	9.78716	3.18449
H	-0.12263	10.79441	3.60196
C	4.56303	7.58974	5.21524
H	5.14430	6.73569	5.59669
H	4.16731	8.14480	6.08086
C	-0.48993	9.53998	1.88611
C	6.11328	7.64808	0.11219
C	-1.02990	10.65116	1.03612
H	-1.51891	11.42665	1.64339
H	-1.75406	10.28098	0.29658

H	-0.21083	11.13677	0.47968
C	5.79854	6.48818	-0.61549
C	-0.40380	8.23869	1.39287
H	-0.77998	8.01886	0.39019
C	4.82063	6.60478	-1.60071
H	4.50394	5.70546	-2.13275
C	0.14752	7.18811	2.13232
C	4.18445	7.82069	-1.86911
C	0.20079	5.81388	1.55017
H	1.22992	5.56383	1.23052
H	-0.44756	5.73881	0.66825
H	-0.07727	5.02335	2.25911
C	4.59660	8.96305	-1.17919
H	4.13119	9.92664	-1.40262
C	3.50534	5.04030	6.58553
C	6.04179	10.13739	0.53153
H	5.65705	10.16340	1.56273
H	5.67722	11.04108	0.02554
H	7.14132	10.18785	0.57453
C	3.09020	7.87536	-2.89311
H	2.41392	7.01568	-2.77477
H	3.50209	7.82478	-3.91445
H	2.50356	8.80112	-2.81177
C	3.65895	5.60843	7.85777
C	8.18071	7.18921	4.53688
C	2.77351	6.72619	8.32485
H	3.09069	7.09251	9.30998
H	1.72614	6.39454	8.40952
H	2.77410	7.57775	7.62816
C	7.77934	5.93493	5.02066
C	4.63916	5.07115	8.69580
H	4.77418	5.50136	9.69218
C	7.94829	5.69596	6.38529
H	7.63181	4.73096	6.78445
C	5.42075	3.98015	8.30608
C	8.49737	6.65194	7.24503
C	6.42545	3.37277	9.24106
H	6.60989	4.01302	10.11467
H	7.38816	3.19088	8.73895
H	6.07091	2.39702	9.61045
C	8.91568	7.87297	6.70830
H	9.36109	8.62496	7.36535
C	5.22112	3.43756	7.03359
H	5.81358	2.57489	6.71523
C	8.77230	8.16452	5.35059
C	4.26468	3.94333	6.14962
C	7.19713	4.90207	4.10559
H	7.09284	3.93388	4.60766
H	7.81745	4.74518	3.21083
H	6.19718	5.18365	3.73828
C	4.02162	3.31983	4.81710
H	4.77475	2.56070	4.57593
H	4.02435	4.05192	3.99327
H	3.02922	2.84561	4.77011
C	8.61425	6.37178	8.71392
H	9.21800	7.13284	9.22692
H	9.07083	5.38806	8.89909
H	7.61681	6.35711	9.18257
C	6.48969	5.18089	-0.37246
H	6.69766	5.00363	0.69100
H	7.44966	5.13969	-0.91573
H	5.86493	4.34521	-0.71249
C	5.57747	8.90654	-0.18786
C	9.20264	9.48487	4.78311
H	9.51188	10.17328	5.58040
H	8.39222	9.96347	4.21019
H	10.05073	9.37268	4.08916
C	2.99963	3.63569	-0.61244
C	4.97441	2.83638	1.56681
C	2.08545	2.95031	2.13690
C	3.19829	1.19992	0.44308

4. Supplementary References

S 1 Ghadwal, R. S.; Roesky, H. W.; Merkel, S.; Henn, J.; Stalke, D., Lewis Base Stabilized Dichlorosilylene. *Angew. Chem. Int. Ed.* **2009,** *48* (31), 5683-5686.

S 2 Franz, D.; Irran, E.; Inoue, S., Isolation of a Three-Coordinate Boron Cation with a Boron–Sulfur Double Bond. *Angew. Chem. Int. Ed.* **2014,** *53* (51), 14264-14268.

S 3 Franz, D.; Szilvási, T.; Pöthig, A.; Deiser, F.; Inoue, S., Three-Coordinate Boron(III) and Diboron(II) Dications. *Chem. Eur. J.* **2018,** *24* (17), 4283-4288.

S 4 Gladysz, J. A.; Tam, W., One-flask preparation of analytically pure dipotassium tetracarbonylferrate. *J. Org. Chem.* **1978,** *43* (11), 2279-2280.

S 5 *APEX suite of crystallographic software*, 2015.5-2; Bruker AXS Inc: Madison, Wisconsin, USA, 2015.

S 6 *SAINT and SADABS*, 7.56a and 2008/1; Bruker AXS Inc: Madison, Wisconsin, USA, 2008.

S 7 Sheldrick, G. M. *SHELXL-2014*, University of Göttingen: Göttingen, Germany, 2014.

S 8 Hubschle, C. B.; Sheldrick, G. M.; Dittrich, B., ShelXle: a Qt graphical user interface for SHELXL. *J. Appl. Crystallogr.* **2011,** *44* (6), 1281-1284.

S 9 Sheldrick, G. M. *SHELXL-97*, University of Göttingen: Göttingen, Germany, 1998.

S 10 Wilson, A. J. C.; Geist, V., International Tables for Crystallography. Volume C: Mathematical, Physical and Chemical Tables. Kluwer Academic Publishers (published for the International Union of Crystallography): Dordrecht/Boston/London, 1992; Vol. C, pp Tables 6.1.1.4 (pp 500-502), 4.2.6.8 (pp. 219-222) and 4.2.4.2 (pp. 193-199).

S 11 Macrae, C. F.; Bruno, I. J.; Chisholm, J. A.; Edgington, P. R.; McCabe, P.; Pidcock, E.; Rodriguez-Monge, L.; Taylor, R.; van de Streek, J.; Wood, P. A., Mercury CSD 2.0 - new features for the visualization and investigation of crystal structures. *J. Appl. Crystallogr.* **2008,** *41* (2), 466-470.

S 12 Neese, F., Software update: the ORCA program system, version 4.0. *Wiley Interdiscip. Rev.-Comput. Mol. Sci.* **2018,** *8* (1), e1327.

S 13 Neese, F., The ORCA program system. *Wiley Interdiscip. Rev.-Comput. Mol. Sci.* **2012,** *2* (1), 73-78.

S 14 Perdew, J. P.; Ernzerhof, M.; Burke, K., Rationale for mixing exact exchange with density functional approximations. *J. Chem. Phys.* **1996,** *105* (22), 9982-9985.

S 15 Adamo, C.; Barone, V., Toward reliable density functional methods without adjustable parameters: the PBE0 model. *J. Chem. Phys.* **1999,** *110* (13), 6158-6170.

S 16 Grimme, S.; Antony, J.; Ehrlich, S.; Krieg, H., A consistent and accurate ab initio parametrization of density functional dispersion correction (DFT-D) for the 94 elements H-Pu. *J. Chem. Phys.* **2010,** *132* (15), 154104.

S 17 Grimme, S.; Ehrlich, S.; Goerigk, L., Effect of the Damping Function in Dispersion Corrected Density Functional Theory. *J. Comput. Chem.* **2011,** *32* (7), 1456-1465.

S 18 Weigend, F.; Ahlrichs, R., Balanced basis sets of split valence, triple zeta valence and quadruple zeta

valence quality for H to Rn: Design and assessment of accuracy. *Phys. Chem. Chem. Phys.* **2005,** *7* (18), 3297-3305.

S 19 Andrae, D.; Häußermann, U.; Dolg, M.; Stoll, H.; Preuß, H., Energy-adjustedab initio pseudopotentials for the second and third row transition elements. *Theor. Chim. Acta* **1990,** *77* (2), 123-141.

S 20 Eichkorn, K.; Weigend, F.; Treutler, O.; Ahlrichs, R., Auxiliary basis sets for main row atoms and transition metals and their use to approximate Coulomb potentials. *Theor. Chem. Acc.* **1997,** *97* (1), 119-124.

S 21 Knizia, G.; Klein, J. E. M. N., Electron Flow in Reaction Mechanisms—Revealed from First Principles. *Angew. Chem., Int. Ed.* **2015,** *54* (18), 5518-5522.

S 22 Römelt, M.; Ye, S.; Neese, F., Calibration of Modern Density Functional Theory Methods for the Prediction of 57Fe Mössbauer Isomer Shifts: Meta-GGA and Double-Hybrid Functionals. *Inorg. Chem.* **2009,** *48* (3), 784-785.

S 23 Bjornsson, R.; Neese, F.; DeBeer, S., Revisiting the Mössbauer Isomer Shifts of the FeMoco Cluster of Nitrogenase and the Cofactor Charge. *Inorg. Chem.* **2017,** *56* (3), 1470-1477.

S 24 Neese, F., Prediction and interpretation of the 57Fe isomer shift in Mössbauer spectra by density functional theory. *Inorg. Chim. Acta* **2002,** *337*, 181-192.

S 25 Wolf, A.; Reiher, M.; Hess, B. A., The generalized Douglas–Kroll transformation. *J. Chem. Phys.* **2002,** *117* (20), 9215-9226.

S 26 Harris, T. V.; Szilagyi, R. K., Comparative Assessment of the Composition and Charge State of Nitrogenase FeMo-Cofactor. *Inorg. Chem.* **2011,** *50* (11), 4811-4824.

S 27 Lenthe, E. v.; Baerends, E. J.; Snijders, J. G., Relativistic regular two-component Hamiltonians. *J. Chem. Phys.* **1993,** *99* (6), 4597-4610.

S 28 van Wüllen, C., Molecular density functional calculations in the regular relativistic approximation: Method, application to coinage metal diatomics, hydrides, fluorides and chlorides, and comparison with first-order relativistic calculations. *J. Chem. Phys.* **1998,** *109* (2), 392-399.

S 29 Alecu, I. M.; Zheng, J.; Zhao, Y.; Truhlar, D. G., Computational Thermochemistry: Scale Factor Databases and Scale Factors for Vibrational Frequencies Obtained from Electronic Model Chemistries. *J. Chem. Theory Comput.* **2010,** *6* (9), 2872-2887.
